# Blockchain-Based Access Control Scheme for Secure Shared Personal Health Records over Decentralised Storage

**DOI:** 10.3390/s21072462

**Published:** 2021-04-02

**Authors:** Hassan Mansur Hussien, Sharifah Md Yasin, Nur Izura Udzir, Mohd Izuan Hafez Ninggal

**Affiliations:** 1 Faculty of Computer Science and Information Technology, Universiti Putra Malaysia, Serdang 43400, Malaysia; izura@upm.edu.my (N.I.U.); mohdizuan@upm.edu.my (M.I.H.N.); 2 Institute for Mathematical Research (INSPEM), Universiti Putra Malaysia, Serdang 43400, Malaysia

**Keywords:** blockchain, decentralised storage, data privacy, attribute-based encryption, searchable encryption, access control, chosen-keyword attack, standard adversary model

## Abstract

Blockchain technology provides a tremendous opportunity to transform current personal health record (PHR) systems into a decentralised network infrastructure. However, such technology possesses some drawbacks, such as issues in privacy and storage capacity. Given its transparency and decentralised features, medical data are visible to everyone on the network and are inappropriate for certain medical applications. By contrast, storing vast medical data, such as patient medical history, laboratory tests, X-rays, and MRIs, significantly affect the repository storage of blockchain. This study bridges the gap between PHRs and blockchain technology by offloading the vast medical data into the InterPlanetary File System (IPFS) storage and establishing an enforced cryptographic authorisation and access control scheme for outsourced encrypted medical data. The access control scheme is constructed on the basis of the new lightweight cryptographic concept named smart contract-based attribute-based searchable encryption (SC-ABSE). This newly cryptographic primitive is developed by extending ciphertext-policy attribute-based encryption (CP-ABE) and searchable symmetric encryption (SSE) and by leveraging the technology of smart contracts to achieve the following: (1) efficient and secure fine-grained access control of outsourced encrypted data, (2) confidentiality of data by eliminating trusted private key generators, and (3) multikeyword searchable mechanism. Based on decisional bilinear Diffie–Hellman hardness assumptions (DBDH) and discrete logarithm (DL) problems, the rigorous security indistinguishability analysis indicates that SC-ABSE is secure against the chosen-keyword attack (CKA) and keyword secrecy (KS) in the standard model. In addition, user collusion attacks are prevented, and the tamper-proof resistance of data is ensured. Furthermore, security validation is verified by simulating a formal verification scenario using Automated Validation of Internet Security Protocols and Applications (AVISPA), thereby unveiling that SC-ABSE is resistant to man-in-the-middle (MIM) and replay attacks. The experimental analysis utilised real-world datasets to demonstrate the efficiency and utility of SC-ABSE in terms of computation overhead, storage cost and communication overhead. The proposed scheme is also designed and developed to evaluate throughput and latency transactions using a standard benchmark tool known as Caliper. Lastly, simulation results show that SC-ABSE has high throughput and low latency, with an ultimate increase in network life compared with traditional healthcare systems.

## 1. Introduction

Blockchain technology has gained considerable attention in many industrial and academic aspects. In particular, the merging of blockchain technology and smart contracts has enabled a ubiquitous decentralised interaction of nodes, thereby yielding an applaudable opportunity for certain applications in private and public domains, such as healthcare systems [1], biomedical sciences [2], and smart cities [3]. In healthcare systems, blockchain technology has played a crucial role in transforming network infrastructure into a stable, secure, auditable, and decentralised environment. The sharing of personal health records (PHRs) is vital for diagnosis and disease care to facilitate patients’ treatment by various medical professionals. PHR systems have become the standard technology that handles the proliferation of generated medical records whilst maintaining the required quality of services. Furthermore, potential blockchain technology enablers, such as decentralised networks, transactions, consensus mechanisms, and smart contracts, can improve security and integrity. However, issues that concern blockchain or smart contracts are related to privacy and storage capacity [4,5,6,7]. The development of PHR applications on the blockchain network may enable anyone to access transaction data due to the blockchain’s transparent feature. This feature has raised privacy concerns regarding the Health Insurance Portability and Accountability Act HIPAA requirements and the ability of patients to participate in the publication of their personal information in the blockchain network. By contrast, blockchain requires considerable storage to record whole transactions in the network; such requirement can be a problem for restrictive nodes that send data to the network. Blockchain can ensure that the stored and shared PHRs are not manipulated, unforgeable and verifiable but can effectively suffer from storage requirements of large-scale distributed data [8,9,10].

An important detail to consider is to examine the advantages and disadvantages of using blockchain technology for PHR against a range of perspectives, such as security, privacy and storage capacity. Recent findings tend to resolve the above issues by storing medical databases on offline storage, such as cloud servers, and by setting up an access control scheme to prevent unauthorised users from manipulating data through leveraging the attribute-based cryptosystem [11,12,13] reproxy encryption [14,15] and smart contracts [16,17,18] to control the users’ privilege. At the same time, other researchers have attempted to offload the actual large-scale distributed data into the InterPlanetary File System (IPFS) storage without setting up any enforced cryptographic access control [19,20]. Meanwhile, concepts of outsourced blockchain databases on honest-but-curious third-party storage, such as cloud servers, are considered a double-edged sword technique that resolves the scalability issue. However, it decreases the security level of blockchain against fully decentralised infrastructure and increases the level of single security failure. Simultaneously, the outsourcing of databases on IPFS decentralised storage eliminates the unreliable storage of third parties. Nevertheless, IPFS has a noticeable security flow that anyone with the hash of the file stored therein can easily retrieve it due to IPFS native workflow. In conclusion, the health data generated by patients are not well suitable for being stored in IPFS unless data are encrypted individually prior to outsourcing to the IPFS. Therefore, providing security and privacy to PHR systems with fine-grained access control is essential to support a technique that searches for encrypted data on the IPFS storage.

Cryptographic primitive methods are considered to be the appropriate solution for confidentiality of outsourced data. However, traditional cryptographic primitive methods, such as symmetric-key encryption and public-key encryption, cannot maintain effective access control over outsourced encrypted data. The ciphertext-policy attribute-based searchable encryption (CP-ABSE) has considerable advantages over other searchable encryption schemes in terms of the construction of secure fine-grained access control for outsourced encrypted data. CP-ABSE is a suitable scheme for storing medical data in the IPFS node by enabling fine-grained access control in an encrypted electronic format to control user privilege and support one-to-many scenarios. This scheme can support expressive access policies by determining any access structures. This scheme also provides a high level of data flexibility because the secret keys (SKs) of the data consumers can be generated at once and can be used to decrypt all the reverent ciphertext. On the contrary, traditional encryption schemes require a trusted private key generator (PKG) to initialise and distribute an SK to users. The PKG may cause serious issues with user data ownership, such as key misuse, data leakage, and capability to control their own data, due to its ability to decrypt all outsourced data stored on the server. Meanwhile, the current scheme has suffered from expensive computational operations in its data outsourcing and retrieval aspects. At the same time, a secure and efficient conjunctive keyword search mechanism for CP-ABSE is essential for the exchange of real data to ensure that only authorised entities can access medical data. In addition, most of the current schemes are not considered the adversary model of security resistance against the chosen keyword attack (CKA) and keyword secrecy (KS) in the standard model.

### 1.1. Motivation

The emergence of PHR sharing systems using cloud technology can provide patients with a complete and accurate online personal medical history, which can benefit patients, research institutions, pharmaceutical companies, and the entire healthcare system. Under these circumstances, the patient’s PHRs are often outsourced to the third party, such as the cloud service provider, to achieve resource sharing and reduce the data centre’s maintenance costs. This situation leads to security issues on how to ensure the security, privacy and searchability of PHRs. To overcome this problem, some researchers are attempting to combine searchable symmetric encryption and ciphertext-policy attribute-based encryption. However, this hybrid encryption scheme requires centralised key management in a cloud server, leading to a single point of security failure because the cloud platform may not be credible due to employee corruption or a threat to the authorisation centre. Fortunately, the use of blockchain technology and smart contracts can easily and securely manage key management and distribution. The concept of creating a permanent and decentralised way to store and share files on IPFS can be perfectly aligned with the blockchain for the provision of a decentralisation infrastructure. Additional blockchain features, such as unforgeable and tamper-proofing of stored data, are also an advantage. Therefore, a secure and efficient CP-ABSE must be designed to support a multikeyword search fine-grained access control for PHRs in the blockchain over IPFS storage without relying on a centralised authority by ensuring its resistance to CKAs and keyword secrecy underneath the standard model.

### 1.2. Contributions

To overcome the aforementioned challenge, this study proposes a new lightweight cryptographic concept called smart contract-based attribute-based searchable encryption (SC-ABSE) by combining ciphertext-policy attribute-based encryption (CP-ABE), searchable symmetric encryption (SSE), smart contract, and IPFS storage. The proposed SC-ABSE eliminates the need for trusted PKGs from the system by allowing the data owner to distribute SKs to data users so that they can control their outsourced encrypted data stored in IPFS; such approach would be more effective than the traditional CP-ABSE schemes. At the same time, the smart contract in the blockchain is used to maintain the SK of users, and the problem of key management in the traditional CP-ABE schemes is resolved. SC-ABSE solves the current problems of developing PHR applications based on blockchain technology by designing a secure and efficient authorisation and access control mechanism that allows patients to store their medical records in a decentralised storage repository (i.e., IPFS) whilst preventing unauthorised users from disclosing medical data. The main contributions of this study are as follows:

- The proposed SC-ABSE scheme removes trusted PKGs by achieving high privacy protection of users’ SKs to ensure the consistent confidentiality of outsourced encrypted data in the IPFS storage. Moreover, it supports a secure multikeyword searchable fine-grained access control by providing one-to-many encryption to prevent unauthorised users from disclosing medical data in decentralised IPFS storage.
- The lightweight key generation algorithm is proposed in the SC-ABSE scheme in comparison with other existing schemes due to a reduced number of pairing operations in which it is a more constant secret key (SK). However, the smart contract is used to auto-enforce the synchronisation of nodes. Any modification in user key distributions can be detected automatically.
- The lightweight outsourcing mechanism is proposed in the SC-ABSE scheme by enabling the blockchain node’s user–patient to encrypt their medical data using the advanced encryption standard (AES) and then only encrypt the symmetric key file and the keyword using attribute-based encryption. This approach reduces the computational complexity of the encryption algorithm by turning into constant ciphertext.
- The lightweight retrieving mechanism is proposed in the SC-ABSE scheme by designing a more secure and efficient token generation algorithm in comparison to other existing schemes. This is due to shifting almost all of the computational complexity processes of the pairing operations to the search part of the IPFS storage entity. Generally, medical data decryption does not depend on the number of attributes in the access control policies.
- The proposed scheme is proven to be secure against CKAs and keyword secrecy under the hardness assumptions of DBDH and DL problems, respectively, in the standard model. Moreover, the formal security verification method using the Automated Validation of Internet Security Protocols and Applications (AVISPA) tool verifies that the reinforced security validation of the proposed scheme withstands replay and MIM attacks.
- A series of comprehensive experimental investigations are performed in terms of computational costs, storage costs, communication costs, throughput, and latency transactions, demonstrating that the proposed scheme achieves a higher level of security with less computational complexity costs than other existing state-of-the-art schemes.

### 1.3. Organisation

The remaining sections are organised as follows: The related work is presented in Section 2. The technical preliminaries of the study are described in Section 3. The proposed scheme and its security definition are presented in Section 4. The security analysis of the proposed scheme is demonstrated in Section 5. The performance analysis of the proposed scheme given in Section 6. Lastly, the conclusion and open directions are summarised in Section 7.

## 2. Related Work

### 2.1. Blockchain-Based Searchable Encryption

The searchable symmetric encryption scheme was first proposed by [21] to ensure that the storage server cannot learn any piece of sensitive information by involving a secure and efficient query of keywords associated with encrypted information. The study [22] proposed a new security definition for searchable symmetric encryption without considering the security of token generation and search queries against keyword secrecy. In [23,24], a new searchable symmetric encryption was presented by designing a secure and efficient scheme based on the order-preserving encryption technique. Meanwhile, [25,26,27,28,29] considered a multikeyword retrieval scheme using the AND gate to access various keywords and protect user information and search tokens, thus maintaining security. The major aim of these approaches is to improve the security of SSE schemes in terms of promoting and expanding their capabilities in a multiuser environment. In conclusion, the literature acknowledges that current SSE schemes are unavoidable in terms of security of CKAs and keyword secrecy due to the need for a strict access policy for outsourced encrypted pieces of information. However, the SSE scheme suffers from critical and expensive key distribution problems. Meanwhile, the SSE scheme enables clients to generate a searchable ciphertext stored on a third-party server, and the server is responsible for searching the keywords associated with the ciphertext. Nevertheless, these schemes require a strict access policy to control the search for outsourced encrypted data on the server due to data owners being unable to grant search rights to their data.

Recent interest in searchable symmetric encryption has given a new impetus to the use of blockchain for improving security on cloud computing and health system research [30,31,32]. These studies have constructed a searchable symmetric encryption scheme by leveraging blockchain features, such as transactions, to retrieve the data stored in cloud servers. The proposed scheme allows users to outsource encrypted data securely to honest-but-curious cloud storage and enables data consumers to search and retrieve data. Moreover, [33] introduced a novel Searchain concept based on the blockchain and keyword search system by enabling users to search securely over a set of keywords attached to data stored in a decentralised repository. In addition, [34,35,36] revealed its tremendous benefits from blockchain technology to develop a secure, searchable encryption system to fulfil the security matter of sharing medical data. Moreover, current SSE schemes require crucial and costly key distribution and cannot support one-to-many encryption. As a result, these shortcomings of the SSE cryptographic primitives have become an improper algorithm for being completely implemented as an independent security mechanism for healthcare applications in blockchain technology. 

### 2.2. Blockchain-Based Ciphertext Policy Attribute-Based Encryption

The ideology of attribute-based encryption (ABE) has gained considerable attention from scholars since it was released in 2006 [37] due to its marvelous characteristics that allow robust fine-grain access control over outsourced encrypted data depending on user attributes. CP-ABE is an ABE variant that allows users to develop an access policy strictly for their data by determining the set of attributes [38]. Numerous researchers have contributed towards CP-ABE to strengthen its capabilities in terms of security [39,40,41] and efficiency [42,43,44] and expand its functionality of supporting searchable mechanisms [45,46,47] to become suitable cryptographic primitives for certain system requirements. These schemes tend mainly to improve communication efficiency and reduce the cost of computational user complexity to access the encrypted data stored in the cloud by utilising the outsourced computation and storage server-aided technique. In addition, user revocation and attribute revocation updates have been achieved, considering the length of ciphertext that could result in high communication costs in practical applications. Simultaneously, the security resistance of these schemes to ciphertext or plaintext chosen attacks is maintained. Lately, several researchers have begun to explore the technology of CP-ABE schemes with medical records to ensure the data security of the exchange of medical data among multiple healthcare providers in a cloud-based environment by developing a secure and efficient system for accessing data [48,49,50,51,52]. The primary objective of these approaches is to obtain a feature of the access structure policy of the CP-ABE and ensure that medical data can only be accessed by matching user attributes. The era of blockchain technology research has moved towards adopting ABE to secure some of its drawbacks in terms of healthcare application [53,54]. These studies have utilised the CP-ABE scheme with blockchain to achieve user authorisation with high flexibility and efficiency for telemedicine systems by utilising the distributed independent key to update the patient keys with multiple healthcare providers to be matched in a real situation.

The conventional CP-ABSE approach presented in Table 1 still needs a trusted PKG. The SK generated by the PKG for users is not adequately flexible. It can lead to misuse of the key by compromising user privacy. To conduct search operations over outsourced encrypted data, these schemes rely on the cloud server. The cloud server can return inaccurate results or even no results to save resources. In comparison, the proposed SC-ABSE scheme uses the IPFS decentralised storage method to resolve a single point of security failure in conventional cloud storage and pressure IPFS to perform the search correctly. Furthermore, the trusted PKG is removed. Another challenge faced by conventional CP-ABSE is the queries of the outsourced encrypted data over the server. The single-keyword search is a trivial procedure in which each keyword is performed separately, resulting in inefficient queries and some information leak to the server. By contrast, multikeyword search allows a user to obtain encrypted data over the sever by attaching several keywords during one single query. Moreover, the proposed SC-ABSE scheme supports secure multikeyword searchable mechanisms without compromising security resistance to chosen-keyword attack (CKA) and keyword secrecy (KS) in the standard model under the DBDH hardness assumption. By contrast, other schemes, [55,56,57,58,59] are analysed in the random oracle model, which is much weaker than the standard model. Such weakness is mainly due to the existence of a trusted PKG, which causes the key escrow problem by compromising the privacy of the user and leads to learning the data user’s search information by testing all token generation and ciphertexts of keywords one by one. However, the CP-ABSE schemes proposed in [55,56,57,58,59] have a high computational complexity that burdens user experience in key generation, token generation, retrieval, and outsourcing of data over cloud storage environments by performing additional computational tasks. This high computational complexity can be a bottleneck for the users to upload and share medical data over an IPFS storage in a blockchain environment. A comparison of these approaches with the proposed scheme in terms of security properties and functionality features is presented in Table 1.

## 3. Preliminaries

### 3.1. Bilinear Groups and Hardness Assumption Problems

For two multiplicative cyclic bilinear groups$\;G_{1}$ and $G_{2}$ of a prime order p, a function $e\;∶\;G_{1}\times \;G_{1}\to \;G_{2}$ is said to be a paired bilinear map if it satisfies the following properties:

- Bilinearity: For all g ∈ $G_{1}$. and a, b  ∈ ${\mathbb{Z}}_{p}$, we have $e\left(g^{a},g^{b}\right)=e\left(g^{b},g^{a}\right)=e{\left(g,g\right)}^{ab}$.
- Nondegeneracy: g ∈ $G_{1}$. exists such that $e\left(g,g\right)\neq \;1$.
- Computability: For any g h ∈ $G_{1}$, an efficient polynomial-time algorithm must exist to compute the pairing $e\left(g,h\right)$.

Suppose B is a probabilistic polynomial-time (PPT) algorithm, where $B\left(1^{n}\right)\to \;\left(n,p,\;G_{1}\;G_{2}\;,e\right)$ and $\left(p,\;G_{1}\;G_{2}\;,e\right)$ are considered an interchangeable variable, and *n* is a system security parameter. Then, this current research uses the hardness assumption problems for β as follows:

The DL problem in $G_{1}$ is defined as follows: given the contrapositive function $B\left(1^{n}\right)\to \;\left(n,p,\;G_{1}\;G_{2}\;,e\right)$ and two consistent elements of g ∈ $G_{1}$ and ∈ ${\mathbb{Z}}_{p}$, the DL assumption states that for any PPT adversary A, a negligible contrapositive function exists such that

(1) $$\mathrm{Pr}\left(\mathrm{adversary}\;\left(n,p,g,g^{x}\;G_{1}\;G_{2},e\right)\;=\;x\;\right)\leq \;\mathrm{negligible}\left(n\right).$$

The above-mentioned probabilistic in Equation (1) is taken upon the random selection of g ∈ $G_{1}$ and ∈ ${\mathbb{Z}}_{p}$. The randomness is used in algorithms B and A, and n is the security parameter of the system.

DBDH problem: Let g  ∈ $G_{1}\;$and α, β, γ ∈ ${\mathbb{Z}}_{p}$ be the four random elements and the contrapositive function of $\left(n,p,\;G_{1}\;G_{2}\;,e\right)$ be an output of $B\left(1^{n}\right)$. The assumption problem of DBDH states that for each PPT adversary A, a negligible contrapositive function exists such that

(2) $$\left|\begin{array}{c} \mathrm{Pr}\;\left(\mathrm{adversary}\;\left(n,p,g,g^{\alpha },g^{\beta },g^{\gamma },g^{\alpha \beta \gamma },G_{1}\;G_{2}\;,e\right)=1\;\right)-\\ \mathrm{Pr}\left(\;\mathrm{adversary}\;\left(n,p,g,g^{\alpha },g^{\beta },g^{\gamma },g^{z},G_{1}\;G_{2},e\right)=1\;\right)\leq \;\mathrm{negligible}\left(n\right) \end{array}\right|$$

The above-mentioned probabilistic in Equation (2) is taken upon the random selection of g ∈ $G_{1}\;$ and α, β, γ, z ${\mathbb{Z}}_{p}$. The randomness is used in algorithms B and A, and n is the security parameter of the system.

### 3.2. Access Control

The access tree’s inner nodes are threshold values, and each leaf node refers to a different set of the attribute. First, assume A is a tree access structure policy, and each nonleaf node is considered a threshold gate value for children’s nodes. The number of children for node x is $Ch_{x}$, and the threshold value is $K_{x}$. However, if the value of $K_{x}=1$, then the threshold is considered an OR gate; if the value of $K_{x}=Ch_{x}$, then the threshold is considered an AND gate that in a matter of the $0<\;K_{x}\leq Ch_{x}$, whereby each leaf node be the representative of an attribute associated with its threshold value of $K_{x}=1$. Afterwards, the parent of node x and the set of attributes associated with the leaf node are denoted as $parent\left(x\right)$ and $\mathrm{attribute}\;\left(x\right)$, respectively. The assigned number of $\left\{1,2,\ldots ,Ch_{x}\right\}$ is an index by node y, where the node y represents a child of node x. These index values are assigned uniquely in an arbitrary manner to the corresponding nodes in terms of the access control structure and are performed as follows:

- If the set of attributes is successfully satisfied with the tree access structure policy A, then $A_{x\;}\left(y\right)=1$.
- If x is a nonleaf node recursively computed on $A_{x^{\prime }}\left(y\right)$ for the entire children $x^{\prime }$ of node x, then $A_{x\;}\left(y\right)$ can return 1 if and only if as a minimum $K_{x}$ children return 1.
- If x is a leaf node, then $A_{x\;}\left(y\right)$ can return 1 if and only if $attributes\left(x\right)\;\in \gamma$.

### 3.3. Functional Encryption of the SC-ABSE Scheme

Assume MD is a message space of medical data, 𝓰 is the access structure space, $U^{s}$ is the space attributes set, S is the space of the attribute set and $\left(\;A\right)$ is the access structure policy. The SC-ABSE scheme consists of six algorithms as follows:

- $\mathrm{Setup}\;\left(1^{k},\mu \right)\to Pk,MK$**.** This algorithm takes a security parameter of $1^{k}$ and a set of universal attributes μ as input. Then, it generates a public key PK and a master key MK.
- $KeyGeneration\;\left(S,MK\;\right)\to SK$. This algorithm takes a defined attribute set $\left(S\right)\;$and MK as input. This algorithm outputs the SK for users in the healthcare user node (HUN).
- $Encryption\;\left(\;PK,MD,kw,\;A\right)\to \left({\mathrm{MD}}^{*},\;SKE_{file},\;I\right)$. This algorithm takes the public key (PK) of users in the HUN, medical data of the patients (*MD*), the keyword (*kw*) and a specified access structure $\left(A\right)$ as input. This algorithm outputs the encrypted medical data (${\mathrm{MD}}^{*}$), the symmetric encryption key for medical data (*SKE*) and the encrypted indexed data (I). Then, it returns the tuple of $\left({\mathrm{MD}}^{*},\;SEK,\;I\right)$ to IPFS storage.
- $GenerationToken\;\left(SK,S,KW\right)\to T\left(T_{kw}S\right)$. This algorithm takes the final secret decrypting keys of users in the HUN $\left(SK\right)$, a set of attributed (S) and given interesting keywords KW as input. Afterwards, the algorithm outputs corresponding token $T\left(T_{kw}S\right)$.
- $Search\;\left(I,T_{kw},\;S,\;A\right)\;\to \left({\mathrm{MD}}^{*}\mathrm{SKE}\right)$. This algorithm takes the given index I with token $\left(T_{kw}\right)\;$and access structure policy $\left(A\right)$ as input. Then, it matches the indexed keywords for the corresponding encrypted medical data with received interested token whatever S (resp., $T_{kw}$) has satisfied with $\left(A\right)$ (resp., I). Then, it sends the encrypted ciphertext of the relevant tuple file results to the requester.
- $Decryption\;\left({\mathrm{MD}}^{*},SKE_{file}\right)\to \left(MD\right)$. This algorithm is takes the relevant search results of encrypted medical data (MD*) and a symmetric encryption key for medical data ($SKE_{file}$) as input. Then, the requester recovers the message $\left(MD\right)$.

## 4. Proposed SC-ABSE Scheme

Here, the typical overall system model for data format and access control protocol is described. Then, the security definition and construction of SC-ABSE are given.

### 4.1. System Model

The proposed SC-ABSE scheme for secure IPFS medical data sharing consists of five entities. These entities are described in Table 2. 

**Figure 1:**
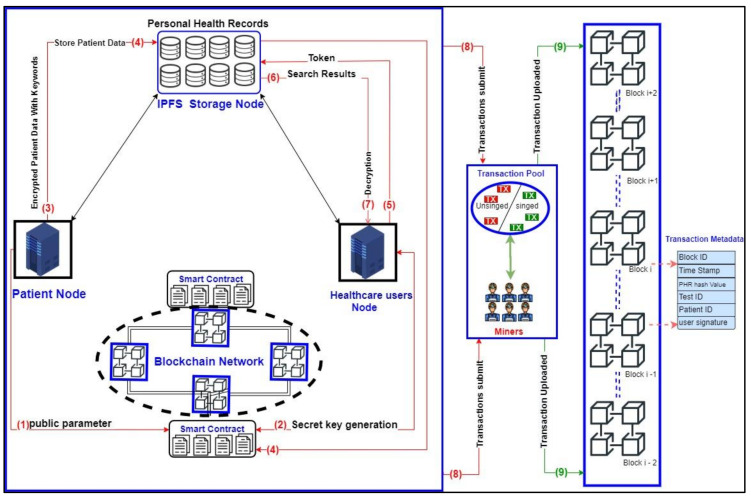
Proposed scheme architecture and system design.

The structure of the proposed SC-ABSE scheme is exhibited in Figure 1, where each step number is described as follows:

(1) The patient node (PN) initialises the system by executing the setup algorithm with the aid of the smart contract to generate public parameters, such as PK and MK whilst publishing PK and keeping MK secret.
(2) The PN generates the final SK associated with the attribute set for each user participating in the HUN by using a smart contract to execute the SK generation algorithm.
(3) The patient in the node encrypts his medical data using AES and then encrypts the symmetric key using ABE, generates the keyword for that encrypted data, encrypts it with the ABE algorithm and sends the tuple of the file, including encrypted medical data, the symmetric encryption key for medical data and the encrypted indexed keyword data to be stored in the outsourced IPFS.
(4) Upon receiving the tuple file from the patient, the IPFS stores the file into distributed node storage and returns the hash value for that file in the form of a transaction and adds it into the transaction pool for confirmation by the miners to append the transaction to the blockchain where each node will have that copy of the transaction. Meanwhile, hash values are stored in the form of a distributed hash table (DHT) smart contract.
(5) The users in the healthcare user’s node search the patient ID via a transaction uploaded to the main blockchain network and generate a token by encrypting keywords of interest using the final SK received from the PN to obtain patient data and then send the attributes and token to the IPFS node.
(6) Upon receiving the token and attributes from the healthcare users, the IPFS searches the keywords if the attributes and token have been satisfied with an access policy and index keywords, respectively. Then, the IPFS sends the tuple file to the users.
(7) After receiving the tuple, the user in the HUN must derive the corresponding symmetric key to decrypt patient data.
(8) Unconfirmed transactions are uploaded for validation by the miners.
(9) Each transaction is signed and appended to the main blockchain network.

### 4.2. Security Requirements and Threats

The security considerations of the proposed SC-ABSE scheme are described in this section.

1. CKA resistance: The proposed scheme can prove its security resistance by formalising the CKA’s provable security indistinguishability. Assuming that the adversary is an IPFS storage, it can access any structure policy throughout the decryption process but cannot obtain information about the actual medical data stored there.
2. Keyword secrecy resistance: It ensures that malicious users in the HUN cannot compromise anything from the patient’s medical data attached to the keyword or token search mechanism.
3. Tamper-proof resistance: The proposed scheme stores medical information, such as diagnostic results or patient history in the IPFS. The adversary intends perhaps to either alter some of the data or replace the existing with another data.
  User collusion attack resistance: SKs for users of the HUN are generated by the PN. Adversary users can generate a token by combining collusively with each other using their own SKs, attempting to retrieve patient data stored in the IPFS.
4. Replay and man-in-the-middle (MIM) attack resistance: Transactions are issued by the PN when the patient users upload data to the IPFS node. An adversary may copy an authorised user transaction from the blockchain or retrieve messages sent by an authorised user. Then, replay or MIM attacks occur when an adversary can change the communication messages on the PN to obtain the patient data stored in IPFS.

### 4.3. Smart Contracts

**Figure 2:**
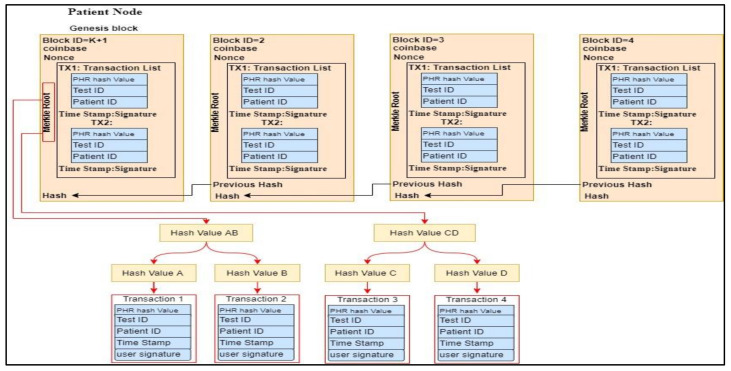
Data block structure.

**Figure 3:**
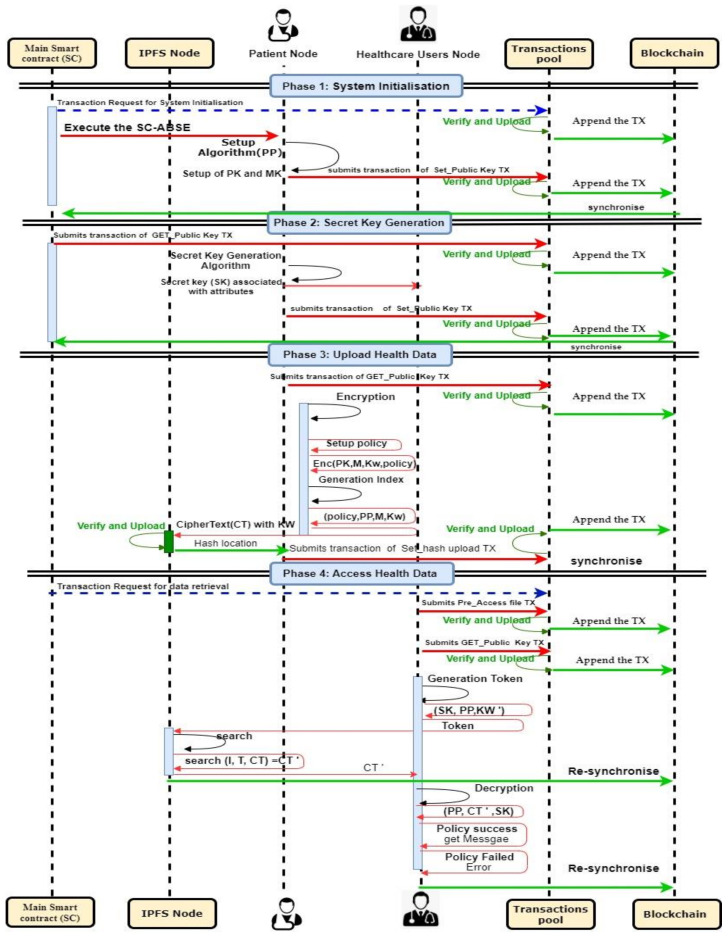
Workflow of generating the smart contract.

The smart contract is an entirely trusted component of the system and is a codable program that lives at the top of the blockchain. It can auto-enforce the code and perform the proposed SC-ABSE scheme managed by a P2P network of nodes. The transaction data structure for the contract is shown in Figure 2. Fundamentally, the access control mechanism is constructed on the basis of the proposed SC-ABSE cryptosystem, and it has four phases. In the system initialisation phase, public security parameters for the PHR system are generated. In the SK generation phase, a request for an algorithm of an SK is held to generate the final SKs for the users in the HUN. In the upload health data phase, the encrypted medical data are outsourced to the IPFS storage. In the access health data phase, a token and search and decryption process is constructed for the medical data stored in IPFS storage. The concrete construction of the above-mentioned phases has been described in detail in Section 4.5. The sequence diagram in Figure 3 specifies how the PN creates the contract. Then, the users in the PN, followed by users in the HUN, consents to the contract. The primary mechanism for the smart contract pattern is explained as follows:

- The PN creates the main contract to execute the SC-ABSE and generate public parameters (PK, MK and SK) for users in the HUN.
- The main contract requires the users in the PN to perform encrypted index keywords and store patient medical data into the IPFS.
- For users in the HUN side, when they wish to retrieve outsourced data in IPFS, the main contract can perform an aspect of the token generated, searched, and decrypted.

### 4.4. Security Model of SC-ABSE

The proposed scheme needs to satisfy the following security requirements: (1) selective security against CKA and (2) keyword secrecy. The following security game experiment is performed on the interactive play between the adversary (A) and the challenger (C).

#### 4.4.1. Chosen Keyword Attacks

On the basis of the security model in the study [60], the provable security indistinguishability experiment CKA is formalised as $SC-ABSE_{A,\;\Pi }^{SCKA}\left(n\right)=1$ by the next security game.

1. Setup: The C executes the Setup algorithm in the system initialisation phase of the proposed scheme to generate public and master keys. Then, the C sends the public keys to the A and keeps the master keys secret.
2. Query Phase 1: The C establishes the list of the keyword list $L_{KW}$, whereas all the lists are initially empty. In addition, the A receives a response as a polynomial number of queries from the C as follows:
  - SK generation query $\left(A_{sk}\right)$: The C executes the KeyGeneration algorithm to obtain SK for users in the HUN, where $A_{sk}$ is a set of attributes if $A_{sk}\notin \;A;\;$then, the C outputs ⊥.
  - GenerationToken Query $A_{sk,kw}$: The C executes the GenerationToken algorithm to generate T (Token) and send it to the A. Finally, if $A_{sk}$ is satisfied with the access structure policy A, then the C adds the keyword to list $L_{KW}$.
3. Challenge: The A chooses two equivalent keywords $\left(kw^{1},kw^{2}\right)$ in which that $\left(kw^{1},kw^{2}\right)$ ∉ to the list of the keywords $L_{KW}$. Afterwards, the C chooses $b\;\in \;\left\{0,\;1\right\}$ and returns over execute the GenerationToken algorithm to produce a keyword index and send it to A. Finally, the C sets $I_{b}\;$=Encryption and sends it to A.
4. Query phase 2: It is a comparable to query as phase 1. However, the A may continue query towards GenerationToken Query $\left(A_{d,\;kw}\right)$ keyword except if $A_{d}\;\in T$. Otherwise, the A is incapable of querying.
5. Guess: The A guesses $b\;of\;b^{\prime }$, where $b\in \;\left\{0,\;1\right\},\;$and it has to be returned by the A. The A wins the experiment game only if $b=\;b^{\prime }$, where the output of the game is defined to be 1. Then, we can write $SC-ABSE_{A,\;\Pi }^{SCKA}\left(n\right)=1$. If the output of the game is 0, then the adversary loses the game.

**Definition** **1.** *The proposed scheme can be secure against the indistinguishability of CKA via Equation (3) if all PPT adversaries had a negligible function.*

(3) $$\mathrm{Pr}(SC-ABSE_{A,\;\Pi }^{CKA}\left(n\right)=1\;\leq \;1/2\;+\;negligible\;\left(n\right)$$

#### 4.4.2. Keyword Secrecy

The provable security experiment of keyword secrecy is formalised as $SC-ABSE_{A,\;\Pi }^{\mathrm{ks}}$. The security game of the keyword secrecy (KS) can be proven via the next experiment [60].

1. Setup: The C runs the Setup algorithm in the system initialisation phase of the proposed scheme to generate the public and master keys. Then, the C sends the public keys to the A and keeps the master keys secret.
2. Query Phase 1: The A issues the below algorithms in polynomial times.
  - *SK generation query*$\left(A_{sk}\right)$: The C runs the KeyGeneration algorithm to obtain SK for users in the HUN, where $A_{sk}$ is a set of attributes if $A_{sk}\notin \;A$. Then, the C outputs ⊥. Otherwise, the C inserts keywords into the list of established medical data queries.
  - *GenerationTokenQuery*$\left(A_{sk,\;kw}\right)$: The GenerationToken algorithm is run by the C to generate T by giving the SK and set of kw. The C sends the token to the A only if the $A_{sk}$. attribute set satisfies the access structure policy. A.
3. Challenge: The A selects the SK and passes it to the C, whereas the C must choose the keyword set $\left(kw^{\prime }\right)$ from the medical data space and run the Encryption algorithm to send the index I to the A.
4. Guess: The A guesses the distinguishable keywords by outputting the keyword set $\left(kw^{\prime }\right)$. The A can win a security game by defining the following $SC-ABSE_{A,\;\Pi }^{\mathrm{ks}}=1\;$experiment if and only if $\left(kw^{\prime }=kw\right)$.

**Definition** **2.** *The proposed scheme can be secure against the indistinguishability of keyword secrecy attack if for all PPT adversary A and the advantage of the adversary to breaking the above keyword secrecy game is at most*$SC-SABE_{A,\;\Pi }^{ks}=\left(n\right)$*, and it has negligible probability in security parameter*n.

### 4.5. Concrete Construction

The proposed SC-ABSE scheme is built on the base of the schemes of [38] and [22]. The notations used in concrete construction are presented in Table 3. The concrete construction of SC-ABSE is described in the following four phases:

#### 4.5.1. System Initialisation Phase

The $\mathrm{Setup}\;\left(1^{k},\;\mu \right)$ algorithm is executed by the PN in the blockchain-based PHR system to generate public security parameters. It defines the attribute space μ, where any j ∈ μ, and the security parameter $1^{k}$. Given two multiplicative cyclic groups $G^{1}$ and $G^{2}$ of prime order p with generators of $g^{1}$ and $g^{1}$ for the security parameter of $1^{k}$. And then, maps the parameters of bilinear $e\;∶\;G^{1}\;\times \;G^{2}\to \;G^{3}$ as a tuple of ($G^{1}$, $G^{2}$,q, g, e). Afterwards, the two random oracle collision-resistant hash functions $h_{1}:\{0,1)*\;\to \;G^{1}$ and $h_{2}:\{0,1)*\;\to \;{\mathbb{Z}}_{q}$ are defined. Moreover, it randomly selects the element of $\alpha ,\;\beta ,\;\gamma \;\in \;{\mathbb{Z}}_{q}\;$by computing the following exponents as $X=g^{\alpha }\;,\;Y=\;g^{\beta }\;,\;Z=g^{\gamma }$. The PN in the blockchain conclusively establishes the security parameter of the system by publishing the PK (as in Equation (4)) and keeping the MK as a secret (as in Equation (5)).

(4) $$PK\;=\;\left(G^{1},\;G^{2},q,\;g,\;e,\;h_{1},h_{2},X,\;Y,\;Z\right)$$

(5) $$MK\;=\;\left(\alpha ,\;\beta ,\;\gamma \;\right)$$

The PN submits a transaction of $\left(Set_{public\;key}TX\right)$ into the transaction pool to be validated by the miners. Whereby, $\left(Set_{Pk}TX={\;\mathrm{Set}}_{\mathrm{Public}\;\mathrm{Keys}\;\left(\mathrm{PK}\right)}\;,\;1^{k}\right)$ is the format of the above transaction. Once the transaction has been broadcasted on the main blockchain network. The smart contract begins compiling and deploying its particular function, such as generating an SK, encrypted patient medical data, generated tokens, performed searches and decrypted. However, any participating node can submit a ${\mathrm{Get}}_{\left(public\;key\right)}\mathrm{TX}$ transaction to the main network of the blockchain. Then, the main smart contract invokes the setup algorithm to initialise public security parameters $\left(1^{k}\right)$.

#### 4.5.2. Secret Key Generation Phase

The $KeyGeneration\;\left(S,MK\;\right)$ algorithm starts executing via the PN by selecting a set of attributes S to generate the final SK for authorised users in the HUN. The user sends the registration request to the PN node to authenticate the user’s identity and assigns the appropriate attributes $\left(S\right)$ by including the user’s account address to the set of permitted users. The PN first chooses a random element of $r\;\in \;{\mathbb{Z}}_{q}$ and then randomly selects an $r_{j}\;\in \;{\mathbb{Z}}_{q}$ for each attribute, whereby j ∈ to set of the attribute (s). Finally, the PN outputs an SK for different users with Equation (6).

(6) $$SK\;=\left(\;\pi =g^{\left(\alpha \gamma \;-r\right)/\beta },\left\{{\;\lambda }_{j}=\;h_{2}g^{r}{\left(j\right)}^{r\;j}\mu_{j}=g^{r\;j}\right\}j\in S\right)$$

The PN submits the following transaction format $({\mathrm{Set}}_{\sec\mathrm{ert}\;\mathrm{key}}\mathrm{TX}=\mathrm{set}\;\mathrm{of}\;\mathrm{attributes}\;\left(S\right),\;\mathrm{SK}$) into the transaction pool to issue SK attributes for users in the HUN. Afterwards, the PN invokes a key generation algorithm in the main smart contract to generate an SK and sends the keys to the users in a secure channel. Each user has been recorded securely on the blockchain with a defined unique attribute authorisation.

#### 4.5.3. Upload Health Data Phase

$Encryption\;\left(PK,MD,kw,\;A\right)$. The users run the algorithm in the PN to establish the keyword index and the encrypt procedure for their medical record set. The algorithm takes PK and the patient health record file for $File\;=\;\left\{f=MD\right\}$ and the keyword dictionary $KW\;=\;\left\{kw\right\}$ as input. The procedure for encrypting and uploading patient medical data in main smart contracts is described as follows:

- Step 1: The patient users define a keyword as $KW_{file}\;=\;\left\{kw_{I}{}_{1}\;,\ldots ,kw_{I}{}_{j}\;,\ldots ,kw_{I}{}_{m}\;\right\}$ for each health data file MD ∈ f and selects random elements $r_{1},r_{2}\;\in \;{\mathbb{Z}}_{q}$ to generate index I, where $I_{j}\;=\;1$ represents the j − th keyword in $KW_{file}$, where this keyword is embedded in MD.
- Step 2: The patient users randomly select AES symmetric key $SKE_{file}$ from the key space and encrypt each file via Equation (7).
  (7) $$\mathrm{FileEnc}\left(f\right)\;={\mathrm{Enc}}_{\mathrm{AES}}\left\{\;file,SKE_{file}\right\}$$
- Step 3: The patient users protect the keyword in the file $KW_{file}$ and the symmetric key encryption of file $SKE_{file}$ under the access policy structure A. Consequently, the users select the polynomial of $q_{x}$ for every individual node x in the access policy structure A by starting from the root node r in a top-down manner. For every individual node x, the degree $d_{x}$ of the polynomial $q_{x}$ is set as $d_{x}\;=\;k_{x}\;-\;1$, where $k_{x}$ is the threshold value of the individual node x. To define the points of the polynomials $q_{r}$ and $q_{x}$ fully, the root node r and node x need to start these algorithms as $q_{r}\left(0\right)\;=\;r_{2}$ and $q_{x}\;\left(0\right)\;=\;q_{parent\;\left(x\right)}\left(index\left(x\right)\right)$, respectively, and then randomly select the elements $d_{r}$ and $d_{x}$. The set of leaf nodes in A is as $l_{n}$, and the index I along with $KW_{file}$ and $SKE_{file}$ are encrypted by giving the tree access structure A and computing it with $\delta_{i}=\;X^{r_{1}h_{2}\left(kW_{i},ske_{i}\right)}\;$whereby $i\in \left\{1,\ldots ,\;MD_{file}\right\}$. Then, the patient users randomly choose the element of $\alpha ,\;\beta ,\;\gamma \;\in \;{\mathbb{Z}}_{q}\;$by computing the following exponents as $E_{0}=\;X^{r_{2}},\;E_{1}=\;Y^{r_{2}},\;E_{2}=\;Z^{r_{1}}$. Finally, $Protect_{kw_{file},ske_{file}}$ can be performed by Equation (8).
  (8) $$Enc_{A}\left(KW_{file},SKE_{file}\right)=\;\left(\theta_{y}=\;h_{1}{\left(\mathrm{attribute}\left(l_{n}\right)\right)}^{q_{\ln\left(0\right)}}\right)$$
- Step 4: The patient users upload the file tuple, including encrypted health data $MD^{*}$, encrypted symmetric key encryption for medical data ($SKE_{file}$) and the encrypted indexed data I, whereby$\;I=\left\{\left\{\;\delta_{i}\right\},\left\{\theta_{y}\right\},E_{0},E_{1},E_{2}\right\}$ to be stored in decentralised storage. IPFS receives the users’ tuple file and places the file location $h_{location}$ and then returns the hash value of that file to be stored in the DHT of the PN. Then, the PN submits an unconfirmed transaction $\left({\mathrm{Set}}_{\left(hash_{location}\right)}\mathrm{hash}\;\mathrm{upload}\;\mathrm{TX}\right)$ to the miner’s pool for validation purposes and broadcasts it on the blockchain’s main network.

#### 4.5.4. Access Health Data Phase

At this phase, the HUN users need to initialise the following transaction format $\left({\mathrm{Submit}}_{\mathrm{transaction}}={\mathrm{Pre}}_{\left(\mathrm{Access}\;\mathrm{file}\right)}\mathrm{TX}\right)$ to the transaction pool to retrieve the patient file. Once the transaction has been approved, users perform token, search and decrypt processing functions in the main smart contract for the medical data stored in IPFS storage.

$GenerationToken\;\left(SK,S,KW\right)$. The users in the HUN creates a token that intends to search for patient data. They first select a random element $s\;\in \;{\mathbb{Z}}_{q}$ and computes the token $T_{1}=\;\prod_{j}^{t}g^{s{\alpha h}_{2}\left(kw_{i}\right)}$, $T_{2}=\;g^{s\gamma }$, $T_{3}=\;\pi^{s}$. Then, they compute ${\lambda^{\prime }}_{j}=\;\lambda^{s}{}_{j}$, ${\mu^{\prime }}_{j}=\;\mu^{s}{}_{j}$ for each attribute j ∈ S and set the token of the submitted keyword set as $T=\left(s,\;T_{1},T_{2},T_{3},{\left\{{\lambda^{\prime }}_{j},{\mu^{\prime }}_{j}\right\}}_{j\in s}\right)$.

$Search\;\left(I,T_{KW},\;S,\;A\right)$. The IPFS node receives the token and attributes of the users of the HUN. The IPFS searches for keywords if the attributes and tokens are satisfied with the access policy structure embedded into the index keywords and encrypted symmetric key encryption. Then, it returns the relevant tuple file through the following steps:

- Step 1: The leaf node is x, and $j^{\prime }=\;\mathrm{attribute}\;\left(x\right)$ only if $j^{\prime }\in \;S$. Then, it computes $MD_{x}$ with Equation (9). Otherwise, $MD_{x}\;=\;⊥.$
  (9) $$MD_{x}\;=\frac{e\left(\lambda_{j}{}^{\prime },\;\delta_{x}\right)}{e\left(\mu_{j}{}^{\prime },\;\theta_{x}\right)}=\;e{\left(g,\;g\right)}^{rsq_{x}\left(0\right)}$$
- Step 2: The nonleaf node is x, and the arbitrary $k_{x}-size$ set of children $x^{\prime }$ is $kw_{x}$. If the set of children $x^{\prime }$ in node x does not exist, then $MD_{x^{\prime }}\neq \;⊥$. Otherwise, it calls the recursive algorithm to compute the following: $MD_{x}=\;\underset{x^{\prime }\;\in \;MD_{x}\;}{\prod }MD_{x^{\prime }}{}^{\Delta_{i,k\omega^{\prime }{}_{x}}{}^{\left(0\right)}}=\;e{\left(g,\;g\right)}^{rsq_{x}\left(0\right)}$, where $i=index\left(x^{\prime }\right),\;k\omega^{\prime }{}_{x}=\{index\left(x^{\prime }\right):$ $x^{\prime }\in \;kw_{x}\}$.
- Step 3: IPFS verifies if Equation (10) holds, and then IPFS sends relevant results, including encrypted medical data $MD_{kw}$ containing the keyword kw and corresponding symmetric encryption key $SKE_{file}$, to users. Otherwise, it returns ⊥, where $MD_{r}=\;e{\left(g,\;g\right)}^{rsq_{r}\left(0\right)}=\;e{\left(g,\;g\right)}^{rsr_{2}}$.
  (10) $$e\left(\prod_{i=1}^{t}\delta_{i}E_{0},T_{2}\right)=e\left(E_{2},\;T_{1}\right)MD_{r^{e}}\left(E_{1},T_{3}\right)$$

$Decryption\left({\mathrm{MD}}^{*},SKE_{file}\right)$: This algorithm is executed by the users of the HUN in accordance with the returned tuple file from the IPFS to obtain the plaintext of the patient file via Equation (11).

(11) $$\mathrm{FileDec}\left(f\right)={\mathrm{Dec}}_{\mathrm{AES}}\left({\mathrm{MD}}^{*},SKE_{file}\right)\to \left(\mathrm{MD}\right)$$

#### 4.5.5. Correctness

The demonstration of the proposed scheme SC-ABSE correctness is conducted by proving that the search algorithm procedure is correct if the attributes and tokens are satisfied with the access policy structure embedded in the index keywords and the encrypted symmetric key encryption. The correctness of Equation (10) can be verified as follows:

Let

(12) $$e\left(E_{2},\;T_{1}\right)=\;e{\left(g,\;g\right)}^{r\alpha \gamma r_{1}\overset{t}{\underset{i=1}{\sum }}h_{2}{}^{\left(kw^{\prime }{}_{i}\right)}}\;e\left(E_{1},\;T_{3}\right)=\;e{\left(g,\;g\right)}^{r\alpha \gamma r_{2}-\;rsr_{2}}\;M_{r}=\;e{\left(g,\;g\right)}^{rsr_{2}}$$

and

(13) $$e\left(E_{2},\;T_{1}\right)=\;M_{r}e\left(E_{1},\;T_{3}\right)=\;e{\left(g,\;g\right)}^{a\alpha \gamma \left(r_{2}+\;r_{1}\;\overset{t}{\underset{i=1}{\sum }}h_{2}{}^{\left(kw^{\prime }{}_{i}\right)}\right)}$$

Then,

(14) $$e\left(\prod_{i=1}^{t}\delta_{i}E_{0},T_{2}\right)=e(g^{a\alpha \gamma \left(r_{2}+\;r_{1}\;\overset{t}{\underset{i=1}{\sum }}h_{2}{}^{\left(kw^{\prime }{}_{i}\right)}\right)})\;g^{s\gamma },\;=\;a\alpha \gamma \left(r_{2}+\;r_{1}\;\overset{t}{\underset{i=1}{\sum }}h_{2}{}^{\left(kw^{\prime }{}_{i}\right)}\right)$$

Therefore, Equation (10) holds that

(15) $$e\left(\prod_{i=1}^{t}\delta_{i}E_{0},T_{2}\right)=e\left(E_{2},\;T_{1}\right)M_{r^{e}}\left(E_{1},T_{3}\right).$$

## 5. Security Analysis

This section discusses the security analysis of the proposed SC-ABSE scheme to validate its robustness in meeting various security requirements. The analysis of security validation is divided into two parts. The first part conducts a formal security analysis on the basis of the DBDH hardness assumptions and DL problems. The second part utilises AVISPA to verify the security correctness of the proposed scheme.

### 5.1. Semantic Security Proof

The security aspects of the proposed SC-ABSE scheme are established on the basis of the following theorems. The security proofs of theorems 1 and 2 are comparable to the scheme in [61].

**Theorem** **1.** *Suppose the DBDH problem is hard relative to ℬ. In that case, the proposed SC-ABSE scheme, under which access control has been built, is secure against the standard model’s CKA*.

**Proof.**  Let Π be donated to the proposed SC-ABSE scheme and A be donated to the PPT in the $SC-ABSE_{A,\;\Pi }^{\mathrm{CKA}}\left(n\right)$ security game experiment referred to in Section 4.4. Construct a simulator $A^{*}$ an adversary that acts as an adversary trying to solve the DBDH problems. The $A^{*}$ chooses four random elements $\left(n,p,G_{1},G_{2},e,g,g^{\alpha },g^{\beta },g^{\gamma },e{\left(g,g\right)}^{z}\right)$, where $B\left(1^{n}\right)\to (\left(n,p,\;G_{1}\;G_{2}\;,e\right)$ and g ∈ $G_{1}$ and α, β, γ∈ ${\mathbb{Z}}_{p}$, whereas z=α, β, γ is the random element of ${\mathbb{Z}}_{p}$. The capability of $A^{*}$ is to determine the unknown value of z. In the construction of the security game of the SCKA, the $A^{*}$ executes A as follows:

1. Setup: The A selects an access structure policy A and submits it to $A^{*}$. Then, the $A^{*}$ selects a consistent element $h_{2}\in G_{1}$ and presumes $t,s\;\in \;{\mathbb{Z}}_{p}$ by setting the parameter as follows:
  (16) $$\left(g_{0}=g\right),$$
  (17) $$(g_{1}=g^{\alpha }),$$
  (18) $$(g_{2}=g^{t}),$$
  (19) $$\left(h=g^{\beta }\right),$$
  (20) $$(h_{1}=g^{\alpha }g^{-s}=g^{\alpha -s}),$$
  (21) $$h_{2}=e\left(h^{-1},h_{1}\right).$$

The $A^{*}$ sends the $\left(n,G_{1},G_{2},e,g_{0},g_{1},g_{2},h,h_{1},h_{2}\right)$, ${\left\{pk_{j}=g^{sk_{j}}\right\}}_{j\in A}$ to the A, where the SK $sk_{j}\in \;{\mathbb{Z}}_{p}$ has been selected randomly for each j ∈ A. Then, by comparing Equations (4) and (18), the trivial value of $g^{t}$ is found to be equal to β. In addition, presume that for unknown $g_{3}\in \;G_{1}$, $h_{2}=g_{3}h_{1}{}^{\beta =t}$= $g_{3}h_{1}{}^{t}$ in Equation (5) of MK generation, which concludes that the PK has been correctly chosen.

2. Query Phase 1: The A establishes the list of $L_{KW}$, and the $A^{*}$ keeps a list of the $L_{KW}\;$for each data user, whereas all the lists are initially empty. In addition, the $A^{*}$ receives a response as a polynomial number of queries from A as follows:
  - *SK generation query*$\left(A_{sk}\right)$: The A runs the KeyGeneration algorithm to generate SK for the users in the HUN. Then, the $A^{*}$ checks whether the set of an attribute in $A_{sk}$ satisfies policy A or not; if not, then for any attribute j ∈ s, it is set as in Equation (22), and then the A returns $A_{sk}={\left\{Sk_{j}\right\}}_{\;}$**,** where j ∈ to set the attribute (s) towards the $A^{*}$.
    (22) $$sk_{j}=\;h_{2}g^{s\beta =sk_{j}}$$
  - GenerationToken Query $\left(A_{sk,\;kw}\right)$: It is comparable to a query of KeyGeneration. The $A^{*}$ has to generate $A_{sk}={\left\{Sk_{v}\right\}}_{\;}$**,** where j ∈ attributes. The $A^{*}$ needs to select a random element of $s\;\in \;{\mathbb{Z}}_{p}$ by creating the search token via Equation (23) and checks whether the attribute satisfies A or not; if yes, then $A^{*}$ adds the keywords to list $L_{KW}$ by sending to the A.
    (23) $$T=\left(T_{1}=\prod_{j}^{t}g^{s{\alpha h}_{2}\left(kw{\;}^{\prime }{}_{i}\right)},T_{2}=g^{s\gamma },T_{3}=\pi^{s},{\left\{{\lambda^{\prime }}_{j},{\mu^{\prime }}_{j}\right\}}_{j\in s}\right)$$

By recalling Equations (6), (17) and (20), the SK generated in Equation (22), and thus the search token in (23) is valid as follows:

(24) h2gsβ=skj= g3h1β=t=(20)g3 gα−sβgsβskj=g3 gαβskj=(17) g3 gαβskj = gt=βg3 g1tskj=(6)skj

3. Challenge: The A returns two equivalent keywords $\left(kw^{1},kw^{2}\right)$ to the $A^{*}$ in which $\left(kw^{1},kw^{2}\right)$ ∉ to the list of the keywords $L_{KW}$. In addition, the $A^{*}$ selects a random element of $r_{1},r_{2}\;\in \;{\mathbb{Z}}_{q}$ to generate encrypted index I. Then, the $A^{*}$ gives the encrypted index $I\left(r_{1},r_{2}\right)$, keyword health data file $KW_{file}\;=\;\left\{kw_{I}{}_{1}\;,\ldots ,kw_{I}{}_{j}\;,\ldots ,kw_{I}{}_{m}\;\right\}$, and public key PK to the A. Then, the $A^{*}$ must select the random value of $b\;\in \;\left\{0,\;1\right\}$ by assuming that if and only if ${\mathbb{Z}}_{q}=\alpha ,\;\beta ,\;\gamma$ and the element of encrypted index $I\left(r_{1},r_{2}\right)$ in the encryption algorithm presented in Section 4.5.3 are selected randomly and correctly.
4. Query phase 2. The A continues his query to the oracles, and the $A^{*}$ responds to a query that is identical to the procedure in phase 1.
5. Guess: The A guesses $b\;of\;b^{\prime }$, where $b\in \;\left\{0,\;1\right\}$. The simulator $A^{*}$ checks whether $b=\;b^{\prime }$ or not. If yes, then the output is equal to 1; otherwise, the output is 0.

As shown in the challenge step, if ${\mathbb{Z}}_{q}=\alpha ,\;\beta ,\;\gamma$ is selected correctly, then the responses returned to A are valid, and the output is as follows:

(25) $$\mathrm{Pr}\left(A^{*}\left(n,p,G_{1},G_{2},e,g,g^{\alpha },g^{\beta },g^{\gamma },e{\left(g,g\right)}^{\alpha ,\;\beta ,\;\gamma }\right)=1\right)\;=\mathrm{Pr}\;(\;SC-ABSE_{A,\;\Pi }^{\mathrm{CKA}}\left(n\right)=1)$$

On the contrary, if $\left(r_{1},r_{2}\right)$ is a random element of ${\mathbb{Z}}_{q}$, then the keyword is also a random element of $G_{2}$. No leakage of any information occurs on the $kw_{i}$ or the analogue. In addition, the A cannot learn any partial information about i, and the output is as follows:

(26) $$\mathrm{Pr}\left(A^{*}\left(n,p,G_{1},G_{2},e,g,g^{\alpha },g^{\beta },g^{\gamma },e{\left(g,g\right)}^{z}\right)=1)=1/2\right)$$

By combining Equations (25) and (26), however, the assumption states that the DBDH is hard relative to B. Then, a negligible function exists such that

(27) $$\left|\mathrm{Pr}\left(SC-SABE_{A,\;\Pi }^{\mathrm{CKA}}\left(n\right)=1\;\right)-1/2\right|,\;=\;\left|\mathrm{Pr}\left(A^{*}\left(n,p,G_{1},G_{2},e,g,g^{\alpha },g^{\beta },g^{\gamma },e{\left(g,g\right)}^{\alpha ,\;\beta ,\;\gamma }\right)=1-\;\mathrm{Pr}\left(A^{*}\left(n,p,G_{1},G_{2},e,g,g^{\alpha },g^{\beta },g^{\gamma },e{\left(g,g\right)}^{z}\right)=1\right)\right)\right|\leq \;\mathrm{negligible}\;\left(n\right),$$

thus proving the theorem. □

**Theorem** **2.** *Suppose the DBDH problem is hard relative to*B. *In that case, the proposed SC-ABSE scheme, under which access control was built, gains the keyword secrecy (KS) in the standard model*.

**Proof.**  In the $SC-ABSE_{A,\;\Pi }^{\mathrm{CKA}}\left(n\right)$ security game experiment in Section 4.4, let Π be donated to the proposed SC-ABSE and the A and the $A^{*}$ donated to the PPT, whereas the assumption of the problem is DL. The authors presume that the elements of $\left(n,p,\;G_{1}\;G_{2}\;,e,g,g^{kw}\right)$ have been given to the $A^{*}$, where n is the system’s security parameter and $B\left(1^{n}\right)\to \;\left(n,p,\;G_{1}\;G_{2}\;,e\right)$ is the relative function, where g ∈ $G_{1}$, and kw∈ ${\mathbb{Z}}_{p}$ is used. The mission of $A^{*}$ is to calculate the trivial value of kw. The $A^{*}$ executes A in the security adversary model of the keyword secrecy as follows:

1. Setup: The $A^{*}$ chooses universal attribute set and random elements α, β, γ ∈ ${\mathbb{Z}}_{p}$. Then, the $A^{*}$ generates the public parameters of the system $=\;\left(n,G_{1},G_{2},e,g_{0},g_{1},g_{2},h,h_{1},h_{2}\right)$, ${\left\{pk_{j}\right\}}_{j\in A}$, and mk=α, β, γ by executing the $\mathrm{Setup}\;\left(1^{k}\right)\to Pk,MK$ algorithm. Then, the $A^{*}$ gives the pk to A and keeps the MKs secret.
2. Query phase 1: The A makes a query to the oracle *SK generation Query* $\left(A_{sk}\right)$. Then, the $A^{*}$ executes the KeyGeneration algorithm to generate SK for the users in the HUN. Finally, the $A^{*}$ gives SK to the A.
3. Challenge: The A declares that Phase 1 is over. Then, the $A^{*}$ runs the KeyGeneration algorithm to generate SK and selects a random element of $r_{1},r_{2}\;\in \;{\mathbb{Z}}_{q}$ to generate index I. Then, $A^{*}$ sets the keyword by $kw=\delta_{i}=\;X^{r_{1}h_{2}\left(kW_{i},sk_{i}\right)}\;$whereby $i\in \left\{1,\ldots ,\;MD_{file}\right\}$. Then, $A^{*}$ returns the kw, I and the PK to the A.
4. Guess: The A guesses the distinguishable keywords $kw^{\prime }$.

If A wins the game experiment of $SC-ABSE_{A,\;\Pi }^{\mathrm{ks}}$, then the $A^{*}$ can solve the DL problem. In addition, the output of the experiment is as follows:

(28) $$\mathrm{Pr}\left(A^{*}\left(n,p,\;G_{1}\;G_{2}\;,e,g,g^{kw^{\prime }}\right)=kw\right)\geq \mathrm{Pr}\;(SC-ABSE_{A,\;\Pi }^{\mathrm{ks}}=1)$$

On the contrary, if the game experiment is under the hardness assumption of the DL problem is believing to says the security game of $SC-ABSE_{A,\;\Pi }^{\mathrm{ks}}$ is

(29) $$\mathrm{Pr}\left(A^{*}\left(n,p,\;G_{1}\;G_{2}\;,e,g,g^{kw^{\prime }}\right)=kw\right)\leq \mathrm{negligible}\;\left(n\right)$$

By combining Equations (28) and (29), however, the probabilistic of $SC-ABSE_{A,\;\Pi }^{\mathrm{ks}}=1$ is a negligible function in n of the parameter security, thus proving the theorem. □

**Theorem** **3.** *Suppose SC-ABSE* = $\prod \left(X\right)$*commits to enforce that the patient’s medical data stored in the IPFS cannot be interfered with or changed. In that case, the proposed SC-ABSE scheme, under which access control was built, manages to gain tamper-proof medical data stored in IPFS*.

**Proof.**  The proposed SC-ABSE = $\prod \left(X\right)$ scheme gains tamper-proof medical data stored in IPFS from the features of the blockchain. It starts by encrypting personal health data with a traditional symmetric encryption algorithm, such as AES. It then uploads the encrypted medical data to the IPFS storage that cannot be modified because upon receiving the patient’s tuple file, the IPFS has to locate an appropriate location for that file, return the hash value $h_{location}$ of that file to the PN to store it securely, submit the transaction$\;\left({\mathrm{Set}}_{\left(hash_{location}\right)}\mathrm{hash}\;\mathrm{upload}\;\mathrm{TX}\right)$ to the miner’s pool for validation purposes and upload it to the main blockchain network. The moderate characteristics of the tamper-proof blockchain ensure the integrity of the data in the transaction and prohibit tampering and failure at any point unless one has upwards of 51% of the computational power of the entire blockchain network, thus proving the theorem. □

**Theorem** **4.** *Suppose SC-ABSE =* $\prod \left(X\right)$ *can ensure that users in the HUN comply with the access structure to retrieve patient data stored in the IPFS node even if they combine token generation. In that case, the proposed SC-ABSE scheme, under which access control was built, manages to achieve resistance to the collusion attack.*

**Proof.**  The proposed SC-ABSE = $\prod \left(X\right)$ is collusion resistant. In the key generation phase of the proposed scheme, the PN requests that users in the HUN delegate their own private key account to be authenticated to assign appropriate attributes and then add the valid user account address of the medical practitioner to the smart contract collection of authorised users. Moreover, the PN has to select a random element $r\;\in \;{\mathbb{Z}}_{q}$ and then randomly select a $r_{j}\;\in \;{\mathbb{Z}}_{q}$ for each attribute by which j ∈ to set of attributes and insert it into the SK attribute for users that were performed through Equation (6). To validate the users, an appropriate token generation of T must be generated using its own sk so that the IPFS storage node can obtain $T_{1}=\;\prod_{j}^{t}g^{s{\alpha h}_{2}\left(kw{\;}^{\prime }{}_{i}\right)}$, $T_{2}=\;g^{s\gamma }$, $T_{3}=\;\pi^{s}$, where $e{\left(g,g\right)}^{s{\alpha h}_{2}}$. Then, IPFS must perform the search algorithm correctly. Otherwise, if invalid users tend to combine their own private keys to form an SK. Therefore, the IPFS node cannot obtain $e{\left(g,g\right)}^{s{\alpha h}_{2}}$ in which the random elements are inserted in the private keys of users in the HUN, thus proving the theorem. □

### 5.2. Security Validation of the Proposed SC-ABSE Scheme in AVISPA

This section presents the security properties of the proposed SC-ABSE scheme by using the AVISPA tool [62]. AVISPA is an automatic tool for evaluating the feasibility of secure internet protocols and applications. This tool offers an expressive and modular formal language for defining protocols and their security properties by incorporating different back-end automated analysis techniques [63]. Many researchers have widely adopted this automatic security verification to validate the security of their proposed schemes [64,65,66,67]. SC-ABSE was designed and coded by a specific programming language called the high-level protocol specification language (HLPSL) via the animator’s security protocol (SPAN) [68]. AVISPA starts translating the high-level language into the intermediate format’s lower-level language (IF) with the built-in translator features named hlpsl2if. Subsequently, the IF specification executes the four backends of the AVISPA tool, namely, the on-the-fly model checker (OFMC), the constraint logic-based attack searcher (CL-AtSe), the SAT-based model checker (SATMC) and the tree automated protocol analyser (TA4SP). AVISPA can then automatically output the scheme’s analysis result on the basis of whether the security requirements are satisfied or violated. Nevertheless, the SATMC and TA4SP backends are less utilised for security protocol validation due to its built-in features that are incapable of verifying algebraic properties, such as modular exponents and XOR operator. This tool mainly strives to validate the protocol security goals against various active and passive attacks, such as MIM and replay attacks.

**Figure A1:**
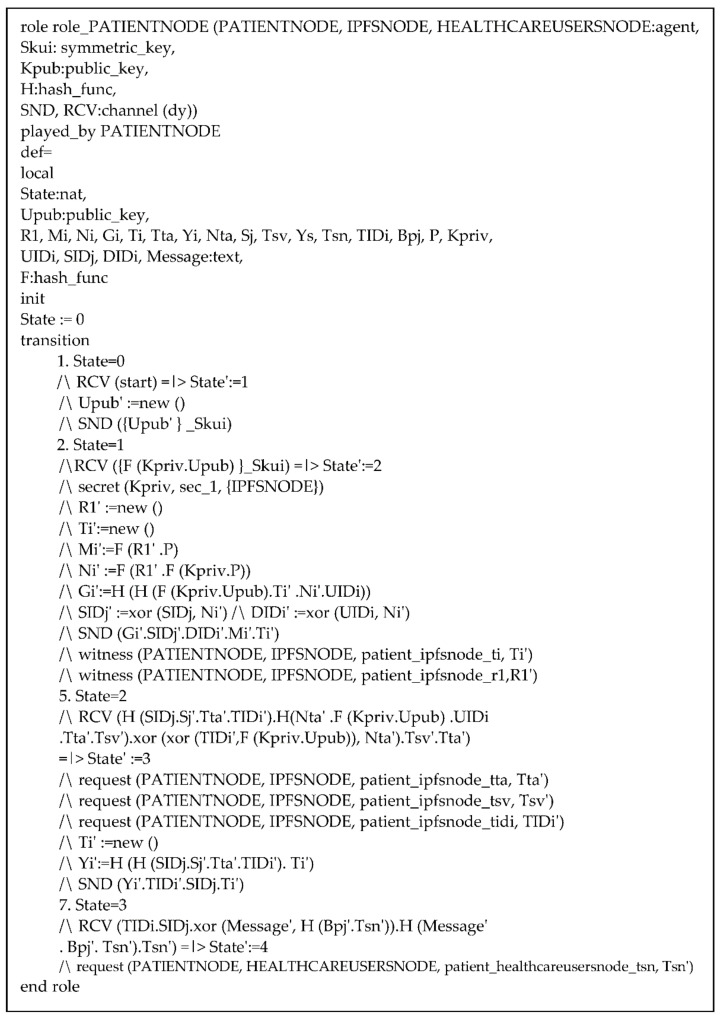
HLPLS specification of the PN role.

**Figure A2:**
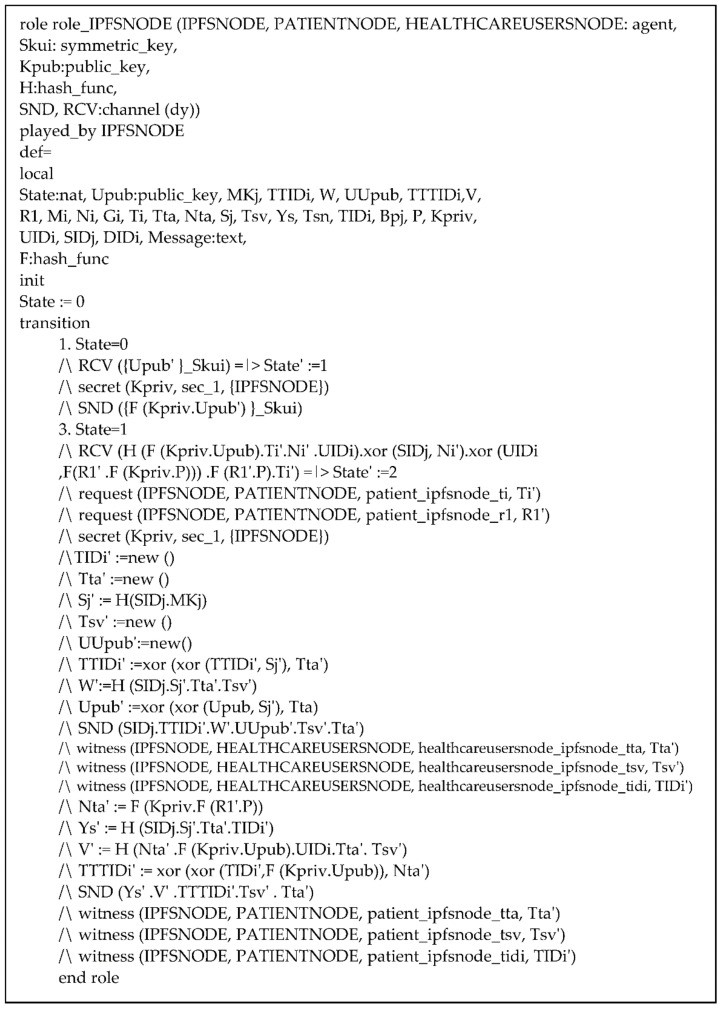
HLPLS specification of the IPFS node role.

**Figure A3:**
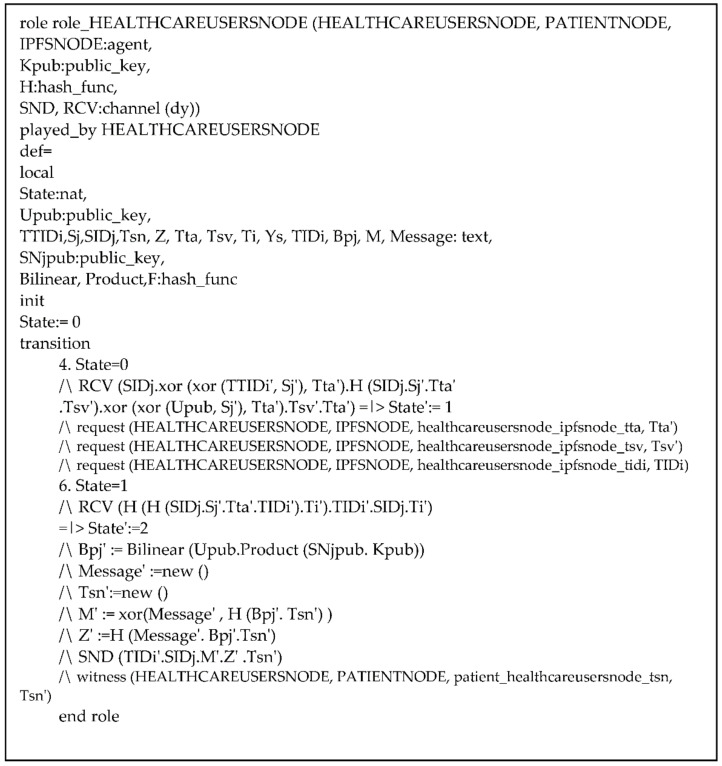
HLPLS specification of the HUN role.

In the security validation of the proposed scheme, the blockchain entity merely establishes and registers each node entity, deploying the smart contract and auditing purposes of all records requests and access activities. The transaction pool contains the unconfirmed transactions of upload and access medical data stored in the IPFS. These two entities considered a constant environment network to deploy such any decentralised applications. Therefore, the security simulation considered three main entities, such as PN, IPFS node and HUN by defining their specifications in the system via HLPLS codes. Moreover, the particular HLPSL code specification, session and environment roles are attached in Appendix A. In Figure A1, the PN enrollment in the blockchain-based PHR is responsible for initialising the system and authenticating each user in the HUN. It also encrypts the medical records and uploads it to the IPFS node. However, the PN needs to communicate with the IPFS node to upload the tuple file $\left(\;MD^{*}SKE_{file}I\right)$. In Figure A2, the users in the HUN need to authenticate the PN to obtain their SK associated with appropriate attributes defined by the users’ enrollment. It also communicates with the IPFS by sending a token associated with an interest keyword to retrieve the tuple file $\left(\;MD^{*}SKE_{file}I\right)$. Figure A3, the IPFS received the encrypted medical data records as a tuple of the file $\left(MD^{*}SKE_{file}I\right)$ need to securely stored file therein. The IPFS also requires to search for the medical records after successfully receiving the token from the HUN. These entities communicate with each other through a secure channel. Substantial consideration must be taken to ensure no secrecy is revealed or passive and active attacks occur.

**Figure A4:**
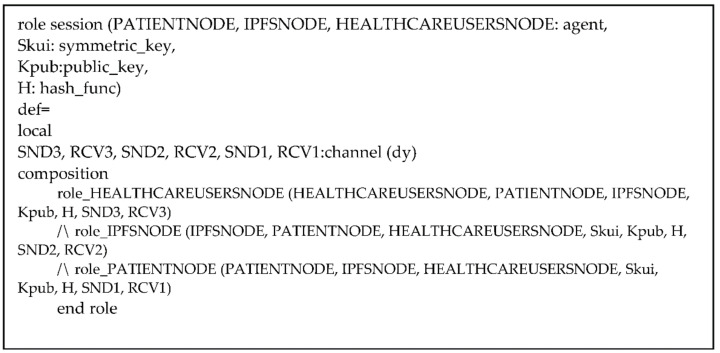
HLPLS specification of the session role.

**Figure A5:**
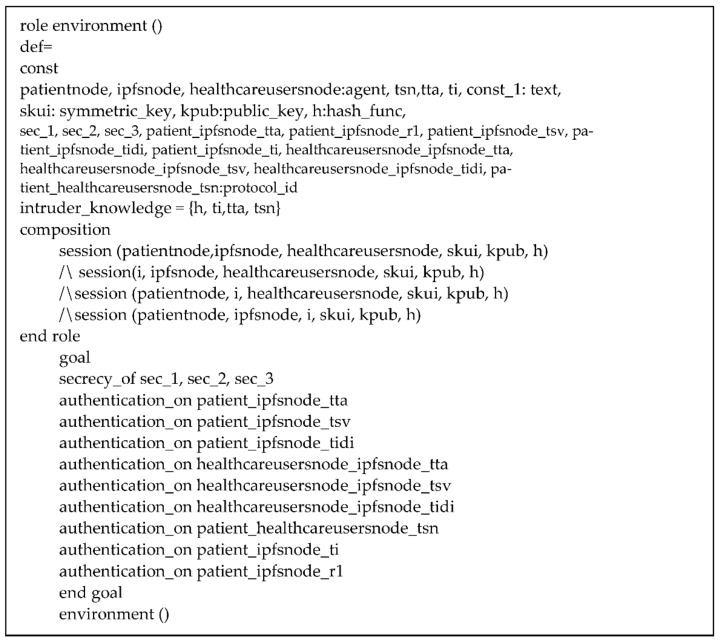
HLPLS specification of the environment role.

In addition, Figure A4 presents the specification of the session’s role for all main entities by defining its composition. The environment’s role is shown in Figure A5 by specifying all essential components within the communication environment, such as symmetric keys, hash function, public key and intruder knowledge. In a simulated environment, the intruder plays a crucial role by replacing the PN, the HUN and the IPFS node roles in the respective sessions in which it intends to compromise the simulated system. The considerations of the secrecy and authentication goals are described in the following aspects:

- *“secrecy_of sec_1”*: The user in the HUN communicates with the PN to obtain the SK and sends it via a secure channel.
- *“secrecy_of sec_2”*: The PN node communicates with the IPFS node and sends a tuple of encrypted medical data in a secure channel.
- *“secrecy_of sec_3”*: The users in the HUN communicates with IPFS and sends a token to IPFS via a secure channel to retrieve the medical data.
- *“authentication_on patient_ipfsnode_tta,tsv,tidi”*: The users in the PN authenticate with IPFS node to upload the file.
- *“authentication_on healthcareusersnode_ipfsnode_tta,tsv,tidi”*: The users in the HUN authenticate with the IPFS node to retrieve the medical data.
- *“authentication_on patient_healthcareusersnode_tsn*”: The PN authenticates the users in the HUN with an appropriate attribute to generate their SK.
- *“authentication_on patient_ipfsnode_ti, r1*”: A request to upload the tuple file is made.

**Figure 4:**
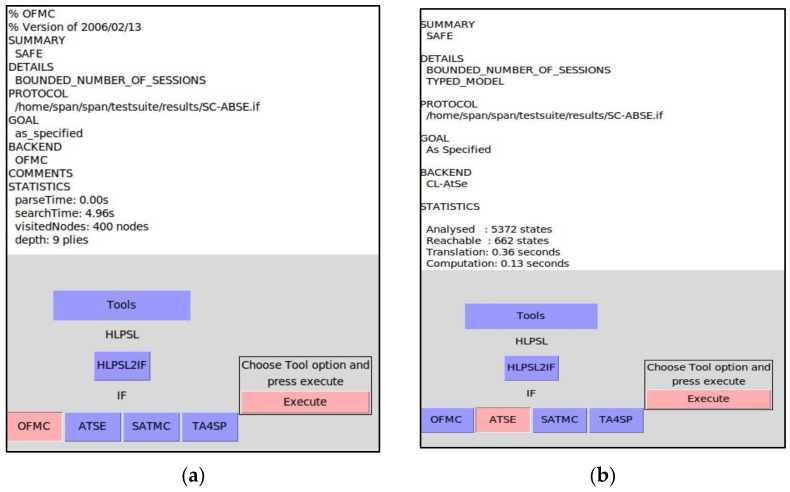
Verification results obtained from AVISPA: (**a**) on-the-fly model checker (OFMC) backend, (**b**) constraint logic-based attack searcher (CL-AtSe) backend.

The proposed scheme is simulated with OFMC, and CL-AtSe backends with a limited range of session model checks after the communication sessions have been established in the environmental role. In the case of a replay attack, the CL-AtSe backend checks whether a legitimate agent can execute the specified protocol by searching for a passive intruder. Afterwards, the intruder shares the knowledge of some normal sessions between the legitimate agents. By contrast, the Dolev–Yao attack model is executed by the OFMC backend to check whether an MIM attack is possible. The intruder may have the possibility of intercepting, analysing and altering messages as long as he knows the required keys and send them to anyone else in the name of any other agent. The present analysis outcome reveals that our scheme could hold out against various attacks, such as replay and MIM attacks, and the intended security objective are all satisfied, as shown in Figure 4. Therefore, the proposed SC-ABSE scheme is secure under AVISPA simulation or its equivalent.

## 6. Performance Analysis

A series of simulations are conducted to evaluate the performance of the proposed scheme. The analysis of performance is divided into two parts. The first part investigates the performance evaluation between the relevant schemes in terms of computational complexity in overhead computation, storage costs and overhead communication. The second part demonstrates the processing time of the smart contracts to deploy the proposed scheme in terms of throughput and latency transaction network.

### 6.1. Computational Complexity Analysis

This section presents a performance analysis of the proposed SC-ABSE scheme in comparison with the existing relevant CP-ABSE schemes. Table 4 lists the notations used in the theoretical asymptotic analysis. The theoretical analysis of computational costs was performed in the context of asymptotic execution complexity. Four types of computational operations, such as paring operation$\;p^{T}$, exponential operation of $e_{1}^{T}$, $e_{2}^{T}$ in $\left|G^{1}\right|,\;\left|G^{2}\right|$, respectively, and the hash operation$\;h^{T}$ mapping of the element in $\left\{0,1\right\}$ to $\left|G^{1}\right|,\;$were considered for measuring asymptotic execution complexity. In addition, the theoretical asymptotic storage cost was undertaken in three groups$\;\left|G^{1}\right|,\;\left|G^{2}\right|$ and$\;\left|{\mathbb{Z}}_{q}\right|$ based on element size. Experimental analysis was conducted on several tests to evaluate the overall performance. Given that electronic medical datasets are not available to the public, we used real-world Enron datasets, which are widely used in many searchable public-key encryption schemes [69]. The public Enron datasets contain approximately 500,000 e-mails from 150 users distributed in 3500 folders, and its size is approximately 0.5 MB of the message. The experimental simulations using Type-A pairings built over the curve $e\left(f_{q}\right):\;y^{2}+x^{3}+x$ [70]. The group$\;\left|G^{1}\right|,\;\left|G^{2}\right|$ of order p as a subgroup of $e\left(f_{q}\right)$ is a large prime number in the Python pairing-based cryptography (pyPBC) library, where the parameters p=160 bits and q=512 bits [71]. Whereby, the value of the $\left|{\mathbb{Z}}_{q}\right|=160\;\mathrm{bits}$, and $\left|G^{1}\right|=\left|G^{2}\right|=1024\;\mathrm{bits}$. The access control policies are being assumed in the AND gate configuration. The experimental workstation is implemented on an Ubuntu 18.04.4 LTS with an Intel Core i5 Processor 2.3 GHz and 8.00 GB. We choose 10,000 files from the public Enron datasets and set the number of attributes [1,50] and perform experimental tests over 100 times in accordance with previous schemes.

#### 6.1.1. Computation Overhead

The theoretical asymptotic analyses of computation overhead for schemes are compared in Table 5. The proposed scheme outperforms the other schemes in terms of execution time overhead to generate a secret key for HUN users in the key generation phase performed by the PN. In addition, the overhead execution time for users in the HUN to generate search tokens for accessing health data phases is remarkably reduced in comparison with [55,56,57]. Moreover, the PN users who generate searchable ciphertext during the upload phase of health data have substantially lower encryption times than other schemes. By contrast, the execution time of the proposed SC-ABSE is higher than that of the scheme in [55] and slightly lower than that of the scheme in [56,57] in the access to health data phase when executing the search algorithm in the IPFS node. 

**Figure 5:**
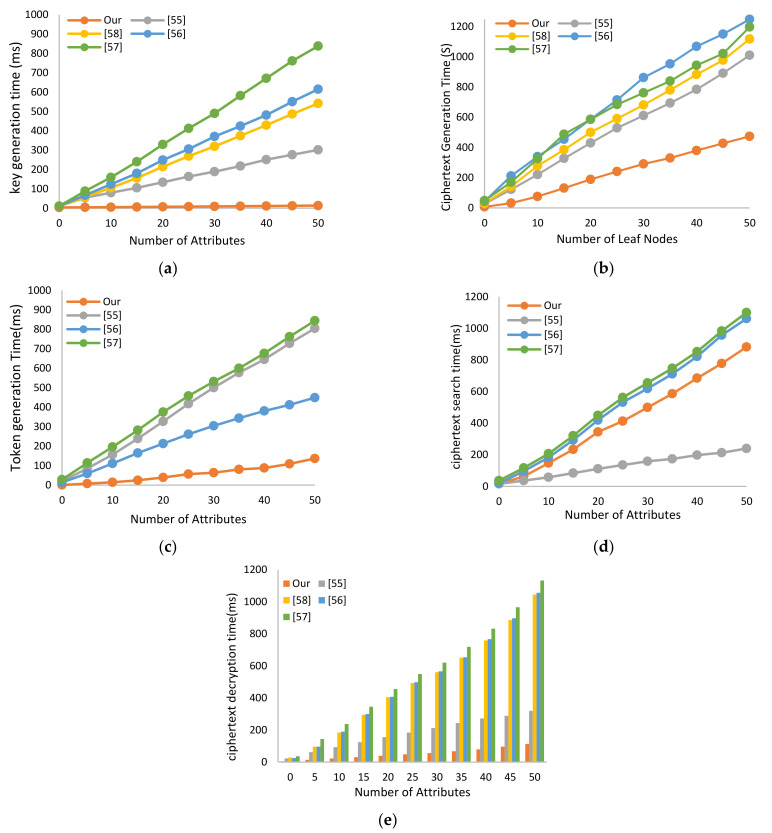
Computation costs: (**a**) execution time for generating users’ SK in the patient node (PN) node; (**b**) execution time for users to generate searchable ciphertext in the PN; (**c**) execution time for users in the HUN to create token search; (**d**) IPFS node to retrieve a requested ciphertext; (**e**) execution time in the decryption algorithm.

The comparison of the actual execution time for the schemes is demonstrated in Figure 5 by setting the number of attributes to 50. In Figure 5a, the key generation algorithm of the proposed scheme takes approximately 13.26 ms in $\left|G1\right|$, whereas other schemes require 301.91 [55], 541.32 [58], 615.46 [56] and 838.18 ms [57]. Given the use of a few pairing operations and requires only one exponential operation of $e_{1}^{T}\;$in $\left|G1\right|$. By contrast, other schemes need to map two pairing operations to generate an SK. The encryption time versus access policy for the number of leaf nodes used to generate encrypted medical data for 10,000 files needs approximately 437.84s, whereas the other schemes [55,56,58], and [57] take approximately 1010.95, 1117.80, 1248.17, and 1197.76 s, respectively, as shown in Figure 5b, because the users in the PN encrypt the medical data via the one-time operation encryption algorithm AES and then only encrypt the symmetric key file and the keyword via ABE. In Figure 5c, the computational cost of the token generation algorithm in the proposed scheme needs approximately 136.17 ms, whereas other schemes in [55,56,57] cost approximately 804.96, 448.97 and 843.25 ms, respectively, because the token generation algorithm of the proposed SC-ABSE scheme is not dependent upon the access control policy’s number of attributes. In addition, most of the computational complexity tasks of pairing operations have been transferred to the search algorithm. However, in Figure 5d, the search algorithm’s performance executed in the IPFS node of the proposed scheme costs approximately 881.56 ms. By contrast, the search time for the schemes in [55,56,57], is 239.32, 1060.95 and 1099.71 ms, respectively. Figure 5d plots the execution time of the decryption algorithm in the proposed SC-ABSE scheme, which needs approximately 113.03 ms, whereas the schemes in [55,56,58], and [57] cost approximately 319.03, 1044.46, 1055.22, and 1132.92 ms, respectively. Given that medical data decryption does not depend on the number of attributes in the access control policies. By contrast, other schemes need to map two pairing operations based on the number of attributes embedded into encrypted medial data.

#### 6.1.2. Storage Cost and Communication Overhead

The proposed SC-ABSE scheme is superior to other schemes in terms of storing the SKs of users in the HUN. In addition, the cost of storing one searchable ciphertext in the IPFS node is remarkably decreased by more than half compared with the other schemes. The communication overhead analysis for transferring the search token from users in the HUN to the IPFS node is dramatically reduced in the proposed SC-ABSE scheme in comparison with others. Therefore, these facts can also be obtained from the data presented in Table 6.

**Figure 6:**
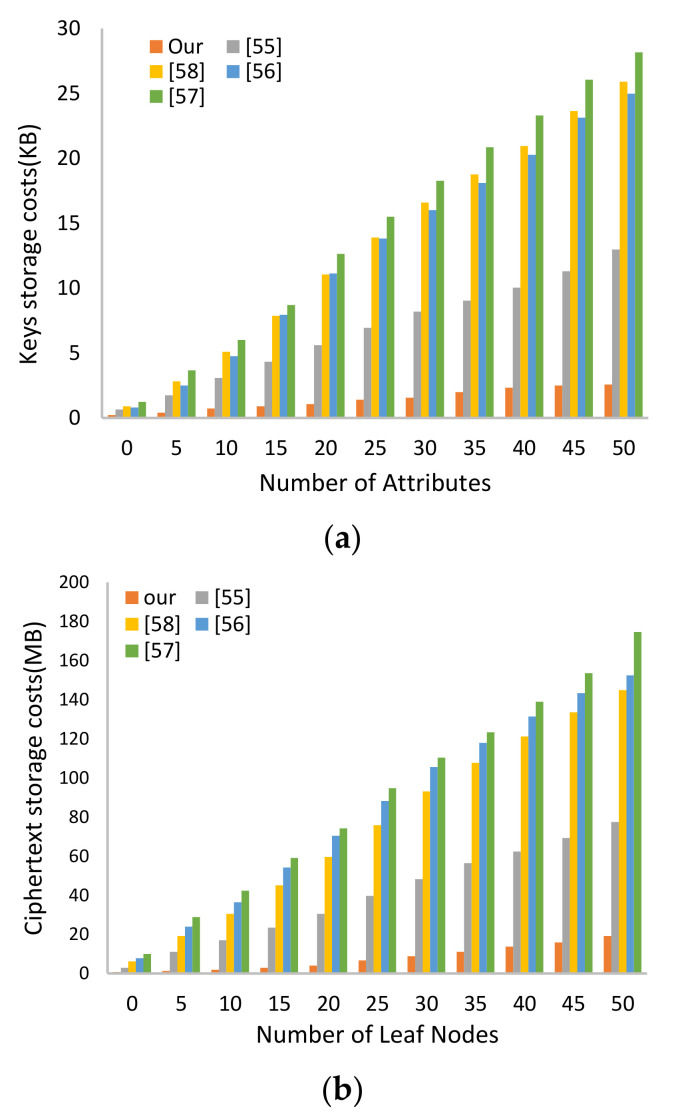

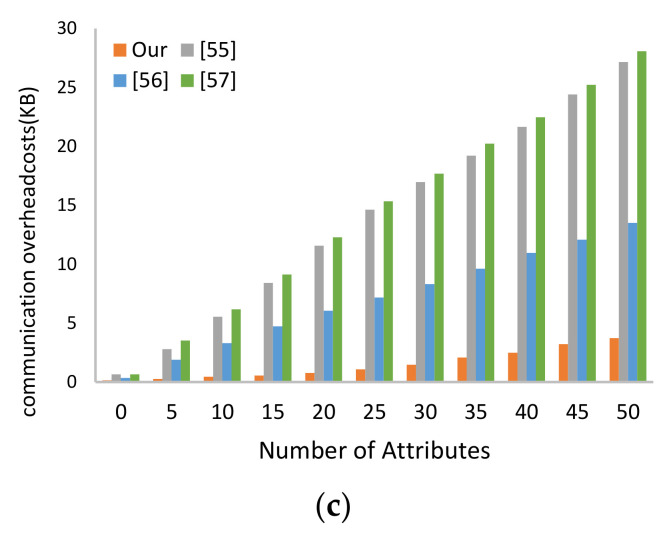
(**a**) Storage overhead for storing the SKs of users in the HUN; (**b**) storage overhead for storing one searchable ciphertext in the IPFS node; (**c**) communication overhead for transferring a search token from the users in the HUN to the IPFS node.

Figure 6 presents the actual performance by setting 50 attributes. In Figure 6a, the storage cost of the SKs of users in the HUN is approximately 2.57 KB, whereas those of the schemes in [55,56,58], and [57] are 12.97, 25.89, 24.97 and 28.15 KB, respectively. In the IPFS node, storage overhead for storing one searchable ciphertext requires approximately 19.08 MB. By contrast, the other schemes [55,56,58], and [57] require approximately 77.43, 144.91, 152.47, and 174.59 MB, respectively, as shown in Figure 6b. In addition, the communication overhead for transferring a search token from the users of the HUN to the IPFS node is approximately 3.71 KB. Conversely, the other schemes in [55,56,57], cost approximately 27.14, 13.49, and 28.07 KB, respectively, as presented in Figure 6c.

### 6.2. Blockchain Network Simulation

This section summarises the proposed scheme’s findings deployed on the blockchain network simulation in terms of transaction throughput and transaction latency metrics.

#### 6.2.1. Simulation Setup

**Figure 7:**
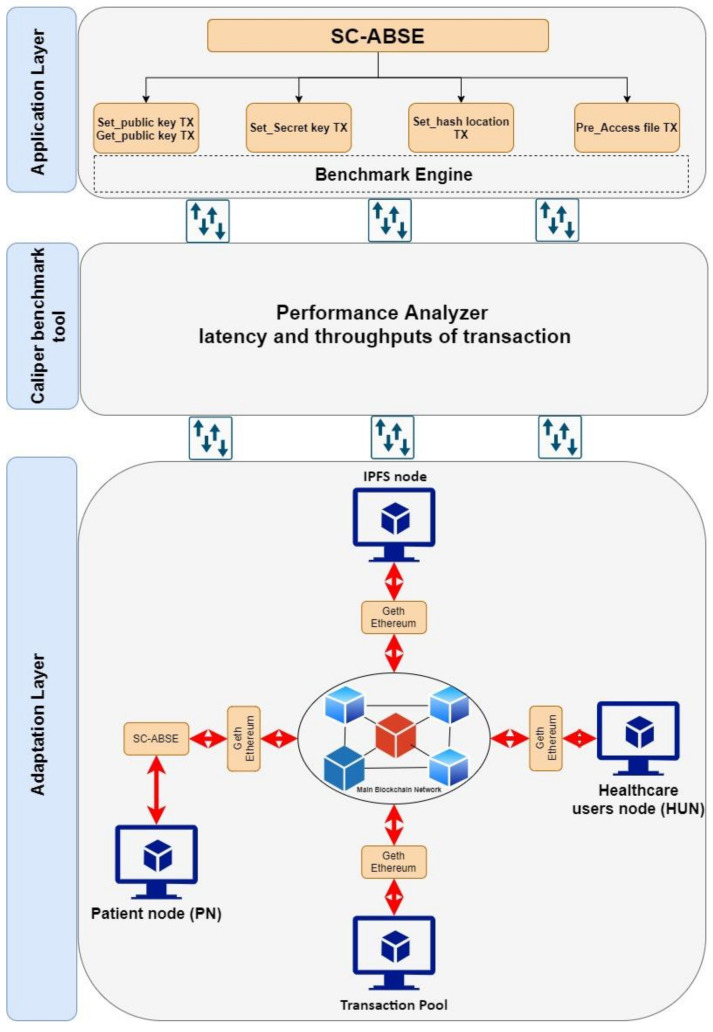
Structure of the blockchain network simulation.

The structure of the blockchain network simulation is shown in Figure 7. The network simulation was set up with four nodes on the basis of the Ethereum blockchain docker [72]. All these nodes have been connected to each other on a different virtual machine. Each machine has an Ubuntu 14.04 LTS operating system with 1.6 GHz vCPUs and 2 GB of RAM. The Clique proof-of-authority consensus protocol^7^ was used to validate and sign the transaction between the node [73]. However, the consensus mechanism is not in the scope of this discussion. The proposed SC-ABSE scheme alongside with the traditional CP-ABSE approach, which are presented in [57], were coded and designed in a smart contract with the help of the Eth-crypto library [74]. Solidity programming was used to design the smart contract’s internal functionality to be deployed on the blockchain simulation platform. The Caliper benchmark tool was used to measure the transaction latency and transaction throughput to determine the schemes’ performance [75]. The specification for the simulation setup is presented in Table 7.

The experiment was performed in a range of 100–1000 concurrent transactions in different measurements and executed in three rounds of transaction writing to the ledger network. However, in the default network configuration, each round has a range of 50–250 transactions per second (TPS). The smart contracts have been deployed between all the nodes for this set of experiments. The throughput and latency average were calculated at the end of the third round using the write workload on the basis of the methods invoked in the smart contracts via Equations (30) and (31).

(30) $$\mathrm{Transaction}\;\mathrm{Throughput}=\;\frac{\;\mathrm{Total}\;\mathrm{committed}\;\mathrm{transaction}\;}{\mathrm{Time}\;\mathrm{per}\;\sec\mathrm{ond}\;\left(s\right)}$$

(31) $$\begin{array}{ll} \mathrm{Transaction}\;\mathrm{Latency} & =\;\left(\mathrm{Transaction}\;\mathrm{execution}\;\mathrm{time}*\;\mathrm{Network}\;\mathrm{threshold}\;\mathrm{time}\right)\\ & -\;\mathrm{Transaction}\;\mathrm{invoke}\;\mathrm{time} \end{array}$$

#### 6.2.2. Throughput and Latency Measurements

**Figure 8:**
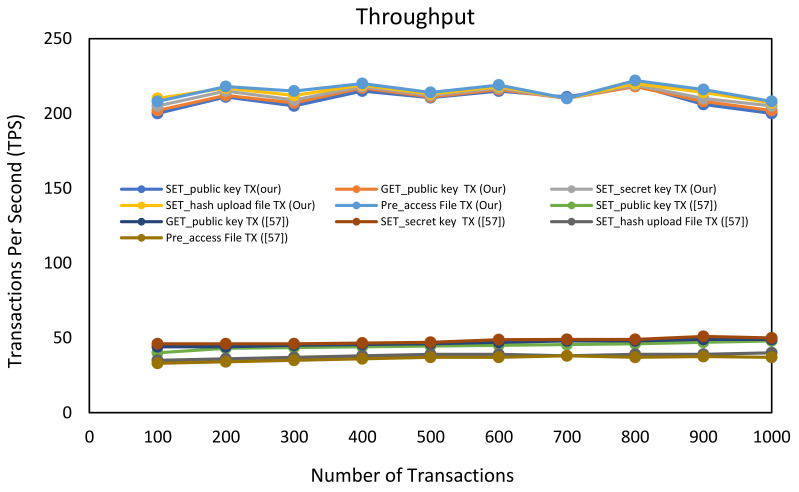
Throughput transaction measurements for smart contract deployments.

Transaction throughput: Numerous TPS have been successfully handled by the blockchain network to be included in the block and to be committed as part of the ledger. The throughput was calculated for the proposed scheme SC-ABSE of blockchain-enabled access control for PHRs in the aspect of involvement transactions for system initialisation, SK generation, uploading of health data, and accessing health data. Figure 8 shows the throughput metric comparison between SC-ABSE and the scheme in [57]. The findings show that the SC-ABSE is comparable for all smart contract settings and has a higher throughput of up to an input load of 200 TPS across the entire range of the network. This can be indicated that SC-ABSE has a good scaling characteristic due to the use of a lightweight cryptographic primitive. Conversely, the scheme in [57] has a fluctuation and a lower rate of throughput transactions of approximately 40 TPS due to the influence of expressive computational operations, as demonstrated in Figure 8.

**Figure 9:**
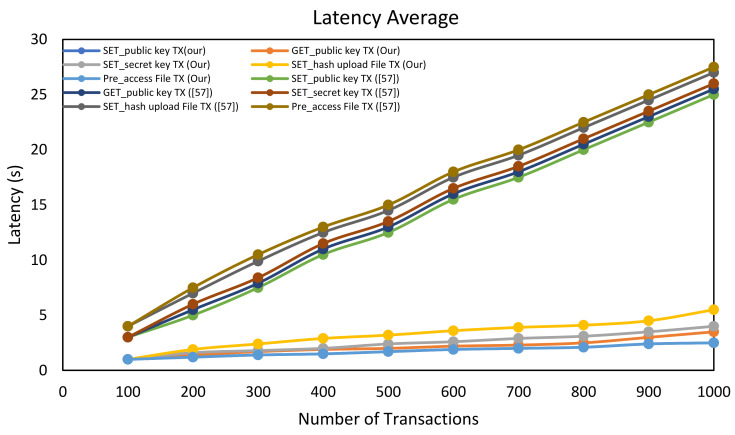
Latency transaction measurements for smart contract deployments.

Transaction latency: The time elapsed between the request and the confirmation event as received by the users after the transaction is confirmed on the blockchain. Figure 9 shows the average latency measurements for SC-ABSE compared with the scheme in [57]. The access control based on the proposed SC-ABSE scheme in terms of system initialisation for the setting up of public security parameters has been approved to commit the 1000 transactions in an elapsed time of 5.5 s due to the lightweight scheme presented in this study. Simultaneously, the medical data’s encryption and storage have tremendously achieved a small scale to conduct 1000 transactions in an elapsed time of 5.9 s. In accessing the health data stored in the IPFS node, the functions used in this transaction are to search, token and decrypt the outsourcing of the data that successfully wrote the transaction in the blockchain network ledger at a rate of 6.9 s of the 1000 transactions committed. This capture results in the simulation of imbalanced times in the transaction when other transactions invoke the smart contract’s internal functionality due to outsourcing the medical data stored in the IPFS, but the underlying trend is approximately unaffected. By contrast, the scheme in [57] has a latency measurement of up to 27 s for 1000 transactions as a comparable average for all settings. If the load has been further continued to increase, the latency average tends to start to degrade mainly due to the increased overhead messaging between the nodes and the cryptographic algorithm involved in encrypting and decrypting the data, thus providing additional computational complexity.

## 7. Conclusions and Open Directions

The present study makes several noteworthy contributions to remedy blockchain technology’s limitations in terms of privacy and scalability storage over healthcare applications. In addition, this study introduces a novel lightweight cryptographic primitive SC-ABSE to secure outsourced encrypted medical data over IPFS storage. This primitive scheme ensures that the patient-user on the blockchain node controls the search for its outsourced encrypted data under the access control policy without the need to rely on trusted PKGs. Any central authority is eliminated from the proposed scheme, and a single point of failure in the system is prevented. The authorised users in the HUN can outsource the search operations to the IPFS and pressure the IPFS to perform the search accurately. Moreover, the computational operations undertaken in SC-ABSE by the patient-user are exceptionally efficient by leveraging the symmetric encryption algorithm and reducing pairing operations. Moreover, HUN users’ consumer is efficiently performed in retrieving medical data due to almost all costly computational operations being offloaded into the IPFS node, and users have been left with extremely lightweight operations. Furthermore, the security definitions and its security resistance of SC-ABSE against the CKA and keyword secrecy are undertaken in the standard model. Moreover, the formal verification tool based on AVISPA proves that the proposed scheme is immune to MIM and replay attacks. The performance analysis measured computational costs, storage costs, communication costs, throughput transactions, and latency transactions from theoretical and practical perspectives. The proposed scheme achieves a high level of security with less computation, storage and communication costs compared with other existing state-of-the-art schemes. The investigation of suggests study in the blockchain network simulation analysis reveals that the throughput that optimises 200 TPS and has a transaction latency is approved to commit 1000 transactions in an elapsed time of 5.5 s. These findings practically imply blockchain’s capability and relevance in numerous fields and highlight that blockchain may be the next revolutionary technology to replace the existing healthcare systems.

The generalisability of these results is subject to certain limitations. Firstly, the traditional access control enables authorised users to decrypt the medical data of patients stored in IPFS. However, it hinders first-aid treatment when the patient’s life is threatened because on-site first-aid medical personnel cannot obtain the patient’s historical medical data. To address this challenge, break-glass access control protocol underneath emergency scenarios is needed in SC-ABSE to allow medical personnel to retrieve the patient’s historical medical data stored in IPFS securely and quickly. Secondly, user and attribute revocation mechanisms are needed throughout (BC-ABSE) to revoke or upgrade their attributes in the system. Therefore, future research should concentrate on the limitations described above to meet a proper decentralised healthcare application.

## Figures and Tables

**Table 1:** Comparison of functional and security properties in various schemes.

Functionality Features	[55]	[56]	[57]	[58]	[59]	Current Study
Lightweight key generation	×	×	×	×	×	√
Lightweight outsourcing mechanism	√	×	×	×	×	√
Lightweight token generation	×	×	×	×	×	√
lightweight retrieving mechanism	×	×	×	×	×	√
Multiowner search	×	×	×	×	√	√
Multikeyword search	×	×	×	×	√	√
Fine-grained keyword search	×	×	×	×	×	√
Decentralised storage	×	×	√	×	×	√
Blockchain based	√	√	√	√	×	√
Eliminates trusted PKG	×	×	×	×	×	√
Security Properties						
Keyword secrecy (KS) in the standard model	×	×	×	×	×	√
Chosen-keyword attack(CKA) in the standard model	×	×	×	×	×	√
Man-in-the-middle attack in the AVISPA toolset	×	×	×	×	×	√
Replay attack in the AVISPA toolset	×	×	×	×	×	√
Tamper-proof resistance	×	×	×	×	×	√
User collusion attack resistance	×	×	×	×	×	√

**Table 2:** Entities of smart contract-based attribute-based searchable encryption (SC-ABSE).

Entities	Description
Blockchain (BC)	Establishes and registers each node entity, such as the patient, the HUN and the IPFS on the network. To impose an agreement on each entity node, a smart contract is used for auditing purposes of all record requests and access activities.
Patient Node (PN)	Initialises system security parameters, for instance, public key (PK), master keys (MK), and final SK for the users in the HUN associated with defined attributes set $\left(S\right)$. The patient node users choose an access structure to encrypted personal information and build the corresponding keyword index and upload the result of the ciphertext attached with an index to the IPFS storage.
Healthcare Users Node (HUN)	The users in the HUN require enough attributes to access the outsourcing ciphertext in IPFS by fulfilling the access structure policy.
IPFS Node	Stores the outsourced encrypted medical data of patients
Transactions pool	Contains the unconfirmed transactions for uploading and access medical data stored in the IPFS

**Table 3:** Notation Definitions.

Notations	Explanation
$G_{1},\;G_{2}$	Multiplicative cyclic bilinear groups
$h_{1},\;h_{2}$	Collision-resistant hash functions
g	Generator of the group $G_{1}$
e	Bilinear map
PK	Public key
MK	Master key
SK	Secret key
SKE	Symmetric key algorithm
A	Access structure policy
μ={1, …, n)	A number set of the attribute
$\mathrm{MD}\;=\;\left\{md_{1},\;\ldots ,md_{p}\right\}\;$	Set of medical files
KW	Set of keywords for medical file
$KW_{file}\left\{kw_{I_{1}},\ldots ,\;kw_{I_{m}}\right\}$	Set of keywords included in medical file
$KW^{\prime }=\left\{kw^{\prime }{}_{1};\ldots ,\;kw^{\prime }{}_{t}\right\}$	Set of Submitted keyword for medical file
$I_{j}/j\in \;\left\{1,\ldots ,\;m\right\}\;\;$	j−th $\;\mathrm{keyword}\;\mathrm{in}\;KW_{file}$
$I_{i}^{\prime }/i\;\in \left\{1,\ldots ,\;t\right\}$	i−th $\;\mathrm{submitted}\;\mathrm{keyword}\;\mathrm{in}\;KW_{file}$
$\left|S\right|$	Set of attributes for users in the HUN
T	Token generation query for a conjunctive keyword
I	Index set of conjunctive keywords

**Table 4:** Notations used in asymptotic analysis.

Number	Notations	Explanation
1	$\left|N_{att}\right|$	Number of medical attributes or data users
2	$\left|N_{A}\right|$	Number of leaf nodes in an access tree A
3	$e_{1}^{T}$, $e_{2}^{T}$	Exponential operation time in $G^{1}$ and $G^{2}$
4	$p^{T}$	Pairing operation time
5	$h^{T}$	Hash operation time
6	$\left|G^{1}\right|,\;\left|G^{2}\right|$	Length of an element in $G^{1}$ and $G^{2}$
7	$\left|{\mathbb{Z}}_{q}\right|$	Length of an element in ${\mathbb{Z}}_{q}$
9	SKE	Symmetric key algorithm

**Table 5:** Comparison of computation overhead.

Scheme	Key Generation	Searchable Ciphertext Generation	Search Token Generation	Data Retrieving	Decryption
[55]	$e_{1}^{T}\left(2\left|N_{att}\right|+4\right)+p+e_{2}^{T}$	SKE + $\left(5\left|N_{A}\right|+2\right)h^{T}\left|N_{A}\right|e_{1}^{T}+e_{2}^{T}$	$e_{1}^{T}\left(2\left|N_{att}\right|+3\right)h^{T}\left|N_{A}\right|$	$\left(5p+e_{2}^{T}\right)\left(N_{A}+3p\right)\left|N_{att}\right|e_{1}^{T}$	$\left(e_{2}^{T}+SKE\right)\left(\left|N_{att}\right|e_{1}^{T}\right)+2\left|N_{att}\right|p$
[58]	$e_{1}^{T}\left(2\left|N_{att}\right|+1\right)+p+2e_{2}^{T}$	$e_{1}^{T}\left|N_{A}\right|+\left(2\left|N_{A}\right|+1\right)e_{2}^{T}$	_________	_____________	$\left|N_{att}\right|e_{2}^{T}+3\left|N_{att}\right|p$
[56]	$e_{1}^{T}\left(3\left|N_{att}\right|+2\right)+p+2e_{2}^{T}$	$e_{1}^{T}\left|N_{A}\right|+e_{2}^{T}\left(3\left|N_{A}\right|+1\right)$	$e_{1}^{T}\left(2\left|N_{att}\right|+3\right)\;2e_{2}^{T}$	$e_{2}^{T}+e_{1}^{T}\left(2\left|N_{att}\right|+4\right)p$	$\left|N_{att}\right|e_{2}^{T}+3\left|N_{att}\right|p$
[57]	$e_{1}^{T}\left(3\left|N_{att}\right|+3\right)+p+e_{2}^{T}\left(2+\left|N_{att}\right|\right)$	$e_{1}^{T}\left|N_{A}\right|+e_{2}^{T}\left(2\left|N_{A}\right|+4\right)$	$e_{1}^{T}\left(2\left|N_{att}\right|+4\right)\;2e_{2}^{T}$	$e_{2}^{T}+\left(2\left|N_{att}\right|+4\right)p$	$e_{2}^{T}\left(2+2\left|N_{att}\right|\right)p\;e_{1}^{T}$
Ours	$e_{1}^{T}$($\left|N_{att}\right|+1)$	SKE + $e_{1}^{T}\left(2\left|N_{A}\right|+1\right)h^{T}\left|N_{A}\right|$	$3e_{1}^{T}+1$	$\left(5p+e_{2}^{T}\right)\left(\left|N_{A}\right|+p\right)$	SKE

**Table 6:** Storage costs and communication overhead comparison.

Scheme	Key Length	Searchable Ciphertext Length	Search Token Length
[55]	$\left(\left|G^{1}\right|+\left|G^{2}\right|\right)\left(2\left|N_{att}\right|+3\right)$	$\left(\left|G^{1}\right|+\left|G^{2}\right|\right)\left(5\left|N_{A}\right|+2\right)\left|{\mathbb{Z}}_{q}\right|$	$\left|G^{1}\right|\left(2\left|N_{att}\right|+3\right)\left|{\mathbb{Z}}_{q}\right|\left|N_{A}\right|+$
[58]	$\left|G^{1}\right|\left(2\left|N_{att}\right|+1\right)$ *+* ${\mathbb{Z}}_{q}+2\left|G^{2}\right|$	$\left|G^{1}\right|N_{A}+\left|G^{2}\right|\left(2\left|N_{A}\right|+1\right)$	*_________*
[56]	$\left|G^{1}\right|\left(4\left|N_{att}\right|+2\right)$ *+* ${\mathbb{Z}}_{q}+2\left|G^{2}\right|$	$\left|G^{1}\right|\left|N_{A}\right|+\left|G^{2}\right|\left(3\left|N_{A}\right|+1\right)$	$\left|G^{1}\right|\left(2\left|N_{att}\right|+3\right)2\left|G^{2}\right|$
[57]	$\left|G^{1}\right|\left(4\left|N_{att}\right|+2\right)$ *+* ${\mathbb{Z}}_{q}+2\left|G^{2}\right|\left(2+\left|N_{att}\right|\right)$	$\left|G^{1}\right|\left|(N_{A}\right|+\left|G^{2}\right|\left(2\left|N_{A}\right|+4\right)$	$\left|G^{1}\right|\left(2\left|N_{att}\right|+4\right)2\left|G^{2}\right|$
Our	*(* $\left|N_{att}\right|+G^{1})$	$G^{1}(2\left(\left|N_{A}\right|+1\right)+2G^{2}$	$3\left(G^{1}+1\right)$

**Table 7:** Simulation Setup.

Network Deployment	Specifications
Blockchain network	Ethereum docker container
Consensus protocol	Clique proof-of-authority
Network analyser	Caliper benchmark tool
**Smart Contract Deployment**	**Specifications**
Programming languages	Vscode Solidity 0.8.0
Cryptographic library	Cryptographic Javascript-functions for the Ethereum (Eth-crypto) library

## Data Availability

Not applicable.

## Appendix A

The appendix, which contains a full source code of the proposed SC-ABSE scheme, is written in HLPSL codes and validated in the AVISPA.
